# Filamentation and restoration of normal growth in *Escherichia coli* using a combined CRISPRi sgRNA/antisense RNA approach

**DOI:** 10.1371/journal.pone.0198058

**Published:** 2018-09-11

**Authors:** Andrea Mückl, Matthaeus Schwarz-Schilling, Katrin Fischer, Friedrich C. Simmel

**Affiliations:** 1 Physics Department, Technische Universität München, Garching, Bavaria, Germany; 2 Nanosystems Initiative Munich, Munich, Bavaria Germany; Imperial College London, UNITED KINGDOM

## Abstract

CRISPR interference (CRISPRi) using dCas9-sgRNA is a powerful tool for the exploration and manipulation of gene functions. Here we quantify the reversible switching of a central process of the bacterial cell cycle by CRISPRi and an antisense RNA mechanism. Reversible induction of filamentous growth in *E*. *coli* has been recently demonstrated by controlling the expression levels of the bacterial cell division proteins FtsZ/FtsA via CRISPRi. If FtsZ falls below a critical level, cells cannot divide. However, the cells remain metabolically active and continue with DNA replication. We surmised that this makes them amenable to an inducible antisense RNA strategy to counteract FtsZ inhibition. We show that both static and inducible thresholds can adjust the characteristics of the switching process. Combining bulk data with single cell measurements, we characterize the efficiency of the switching process. Successful restoration of division is found to occur faster in the presence of antisense sgRNAs than upon simple termination of CRISPRi induction.

## Introduction

Before an *E*. *coli* cell divides into two identical daughter cells, proteins of the cell division machinery accumulate at its center with an accuracy of about 2% and form the so-called ‘Z ring’ [1].The Z ring serves as a scaffold at the future division site for the other over 20 known proteins that constitute the divisome [2]. The Z ring itself is assembled from at least six proteins, including the filament forming protein FtsZ and its membrane anchoring proteins FtsA and ZipA.[3–6]. The cellular content of FtsZ needs to be regulated, as under- or over-expression leads to filamentous bacteria or minicells without a genome [7, 8]. Moreover, an imbalance between FtsZ and FtsA results in cell division arrest and bacterial filaments that contain multiple copies of the bacterial chromosome [9, 10]. Since FtsZ is one of the earliest proteins to initiate the assembly of the division machinery, mutations or knock-downs of FtsZ result in filaments with stalled constriction sites or partially divided regions [11]. One of the earliest studied strains with a mutation in *ftsZ84*, displays filamentous growth at 42°C and can be switched back to normal growth at 30°C [12, 13]. Aborted constriction sites do not seem to be continued after temporary upshifts from 30°C to 42°C for 2 minutes, but newly formed division sites are used [14]. However, the *ftsZ84* mutant lyses or loses its viability after about 3 hours after the temperature shift.

Recently, Sánchez-Gorostiaga *et al*. studied the response of *E*. *coli* when FtsZ falls below a critical level, followed by its restoration using an IPTG inducible promoter in front of the *ftsZ* gene [15]. They show that besides forming filaments, FtsZ deprived cells are more prone to improper chromosome segregation, show global changes in transcription levels and lose integrity of their membrane.

A convenient technique for the regulation of chromosomal gene expression has been recently provided through CRISPR interference (CRISPRi) using dCas9, a non-cleaving mutant of the CRISPR associated nuclease Cas9 [16]. The protein can be directed to any position on the chromosome by a single guide RNA (sgRNA) molecule, provided that the target sequence neighbors a protospacer adjacent motif (PAM) with the canonical sequence NGG. Depending on the binding site, the dCas9-sgRNA complex can repress transcription by either preventing transcription initiation by RNA polymerase or by acting as a roadblock for transcriptional elongation.

Elhadi *et al*. have recently used the CRISPRi mechanism to change the morphology of *E*. *coli* by targeting the *ftsZ* gene and the *mreB* gene, a gene found to control the width of the cells [17, 18]. Focusing on microbial bioproduction of plastics, they found that the morphologically altered cells provide a larger volume for the accumulation of intracellular polyhydroxybutyrate (PHB) inclusion bodies. However, the CRISPRi mediated gene knockdown had a negative impact on bacterial growth rate.

In order establish a reversible knockdown system based on CRISPRi, Lee *et al*. utilized antisense RNA to target the sgRNA [19]. They were able to show that the expression of fluorescent proteins could be successfully suppressed and reactivated in bacterial cells, with each step having a response time of ≈ 3 hours. Using RNA instead of proteins to regulate gene expression can have several advantages. They are straightforward to design since RNA-RNA interactions can often be reduced to base-pair interactions and their secondary structure can be predicted with software tools [20, 21]. Moreover, RNA is expressed faster than proteins and requires less of the cell’s resources [22].

In the present work, we reversibly induce filamentation in *E*. *coli* by targeting FtsZ using the CRISPR/dCas9 interference mechanism combined with an antisense RNA strategy. We first switch bacteria into the filamentous cell growth mode and subsequently reverse this process, such that the cells return into a normal growth phenotype. We quantify the switching process and dynamically control the system by the inducible expression of antisense RNAs that are complementary to the sgRNAs engaged in *ftsZ* knockdown. We use single-cell fluorescence microscopy experiments from which we derive bacterial length distributions [23], growth and division rates, as well as reporter gene expression levels. This allows us to identify factors that affect the switching process. In particular, we find that bacteria strongly respond to the CRISPRi knockdown of *ftsZ*, but only few bacteria revert to normal cell division several hours after termination of CRISPRi induction. Antisense-sgRNA expression supported the recovery process and facilitated considerably faster switching than in the absence of the antisense RNAs. Antisense-sgRNA thus provides a relatively straightforward means to control and adjust the kinetics of CRISPRi de-repression, which is of great interest for the realization of gene circuitry, in which cellular processes have to be dynamically switched on or off in response to endogenous signals or external cues.

## Results

### Experimental design with decoy-binding sites

**Fig 1:**
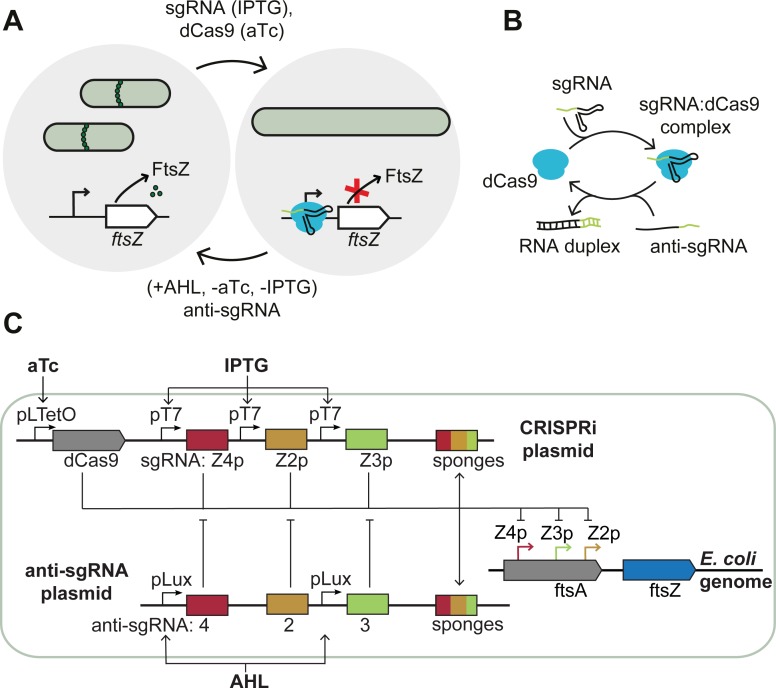
CRISPRi-based growth control. **(A)** Schematic representation of *E*. *coli* switching into filamentous growth after induction of single guide RNA (sgRNA) via IPTG and dCas9 via aTc. The dCas9-sgRNA complex blocks the expression of FtsZ, stopping the formation of the septal ring that is essential for cell division in *E*. *coli*. Cell division can be rescued by inducing appropriate antisense sgRNAs (‘anti-sgRNA’) with AHL and by removing the inducers for the dCas9 and sgRNA. **(B)** Scheme of sgRNA forming a complex with dCas9. The anti-sgRNA can inhibit formation of the complex by binding to the sgRNA. **(C)** Details of the genetic constructs involved: the CRISPRi plasmid codes for dCas9 under aTc-inducible promoters and three different sgRNAs under T7 promoters which target three different promoters of the *ftsZ* gene on the genome of the *E*. *coli*. T7 RNA polymerase is inducible with IPTG. The ‘anti-sgRNA plasmid’ codes for anti-sgRNAs under the control of an AHL-inducible promoter. The sponge elements on the plasmids act as decoy binding sites for the corresponding dCas9-sgRNA complexes.

In order to reversibly switch bacteria to filamentous growth, we disturbed the FtsZ/FtsA ratio through CRISPR interference using appropriate sgRNAs (Fig 1A). Three of at least six known promoters for *ftsZ* (ftsZ2p, ftsZ3p, ftsZ4p) lie within the *ftsA* coding region [24]. We targeted the template strand of these three promoters for *ftsZ* transcriptional initiation blockage (Fig 1B and S1 Table). In contrast to tunable CRISPRi (tCRISPRi) with an inducible chromosome-integrated dCas9 [25], we here use plasmid-encoded inducible dCas9 and sgRNA. The sgRNAs are induced by IPTG (Isopropyl-β-D-thiogalactopyranoside) via T7 RNA polymerase and were encoded on the ‘CRISPRi plasmid’ together with TetR-controlled dCas9 and mVenus reporter protein.

We then restored cell division using different strategies: i) first, by removing CRISPRi inducers aTc and IPTG and thus turning off dCas9 and sgRNA expression, or ii) by inducing anti-sgRNAs, which counteract the CRISPRi mechanism (the ‘antisense’ strategy), or iii) by removing CRISPRi inducers and adding anti-sgRNA inducer (the ‘active’ approach). The anti-sgRNAs were designed to absorb the sgRNAs via duplex formation (Fig 1B) in a similar manner as previously demonstrated [19]. In our approach, the anti-sgRNAs are transcribed from the ‘anti-sgRNA plasmid’ and induced by AHL (acyl homoserine lactone (HSL) 3-oxo-C6-HSL) from pLux promoters (S2 Table).

Transformation of bacteria with the CRISPRi plasmid resulted in filamentous cells even without induction of the expression of dCas9 and sgRNA. In order to create a threshold for dCas9-sgRNA below which *ftsZ* is not regulated down, we introduced decoy-binding sites (‘sponges’) for the dCas9-sgRNA complex. However, the addition of sponges to the CRISPRi plasmid did not stop filamentation in the absence of inducers (Panel A in S1 Fig). Only after the introduction of additional sponge elements on the high copy number plasmid (∼500–700 per cell [26]) that encodes the anti-sgRNA, the bacteria exhibited the intended normal growth morphology (Panel B in S1 Fig and S1 Movie).

Expression of dCas9 in the absence of sgRNA or with sgRNAs of different sequence does not lead to filamentous growth under our experimental conditions (Panel C in S1 Fig). We can therefore rule out that dCas9 alone was responsible for the observed changes in cell morphology, as was reported previously for high level expression of the protein [27].

### Reversal of CRISPR interference using antisense sgRNA

**Fig 2:**
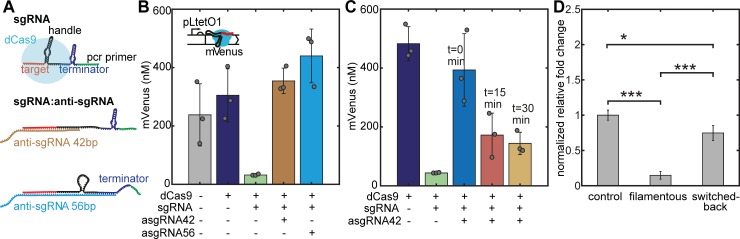
Experimental validation of the anti-sgRNA strategy. **(A)** Secondary structures of free and complexed sgRNA and anti-sgRNA variants. The dCas9 handle is destroyed by duplex formation, which prevents dCas9 from binding. **(B)** Prototyping of the CRISPRi knockdown and rescue system in a cell-free gene expression system using mVenus as a fluorescent reporter protein. The restoration of mVenus expression is shown with truncated anti-sgRNA versions (the number indicates the number of base pairs in the resulting sgRNA:anti-sgRNA duplex). The expression of mVenus ([template DNA] = 5 nM) is blocked by the supplementation of purified dCas9 (70 nM) and sgRNA (100 nM) and is re-activated upon addition of anti-sgRNA (0.5 μM). Fluorescence levels for three different samples are taken at t = 15.5 hours or t = 11 h. Error bars are plotted as SD from 3 individual replicates. **(C)** Delaying the time of anti-sgRNA addition relative to dCas9-sgRNA results in lower mVenus fluorescence intensities. (taken at t = 12 hours). Error bars are plotted as SD from 3 individual replicates (c(sgRNA) = 250 nM, c(anti-sgRNA) = 1 μM). **(D)** Bar plot of RT-qPCR data, showing *ftsZ* RNA levels of normal, filamentous (107 nM, 1 mM IPTG) and switched back (107 nM, 1 mM IPTG, 100 nM AHL) cells from three technical replicates. The asterisk marks indicate the significance levels between the samples.

In order to test the effect of anti-sgRNA on CRISPRi, we initially performed a series of *in vitro* experiments in homemade bacterial cell extract [28] and in a commercial gene expression system (S2 Fig). First, we tested two different designs for the anti-sgRNAs, which differed in their lengths and bound to different complementary regions of the sgRNA, one creating a 42 bp and the other a 56 bp duplex with the sgRNA (Fig 2A). We regulated the expression of mVenus using purified dCas9 together with sgRNA and anti-sgRNA. As shown in Fig 2B (and S2 Fig), mVenus expression is efficiently suppressed in the presence of dCas9 and sgRNA, while the addition of the anti-sgRNA variants recover the production of mVenus (cf. S3 Table for the corresponding sequences). Our experiments confirmed that sequestration of the spacer region and only part of the dCas9 handle by the short anti-sgRNA is sufficient to de-activate the CRISPRi mechanism (Fig 2B). Thus, in our *in vivo* experiments also the short anti-sgRNA design was applied. In a second experiment, we delayed the addition of anti-sgRNA relative to dCas9-sgRNA. We found that mVenus fluorescence is recovered compared to the knockdown case, with intensities decreasing for longer delay times (Fig 2C).

We next characterized *ftsZ* gene expression levels in normal, filamentous and switched-back cells using RT-qPCR. Upon induction of the CRISPRi mechanism the *ftsZ* mRNA level was strongly reduced relative to non-induced normal growing cells. Induction of anti-sgRNA transcription (while maintaining the CRISPRi induction) recovered the mRNA level again (Fig 2D and Panel A in S3 Fig). A western blot test conducted analogously confirmed the varying amounts of FtsZ at the protein level (Panel B in S3 Fig).

### Single-cell analysis of filamentation

**Fig 3:**
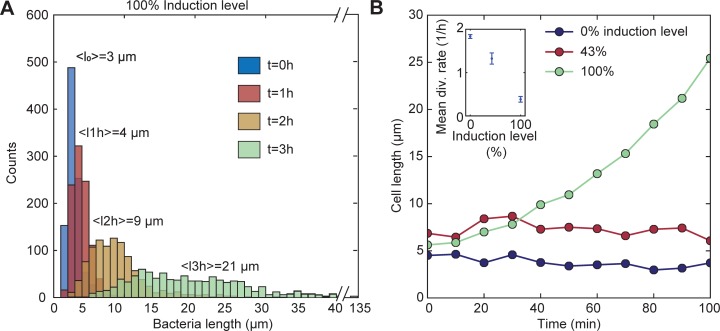
Filamentous bacteria. (**A)** Histogram of the cell length (calculated from 1000 cells each) at different time points at 100% induction level (corresponding to 500 μM IPTG and 107 nM aTc). Over time the distribution broadens and the whole population shifts to larger lengths. **(B)** Tracking the cell length at different induction levels. Inset: division rates decrease with increasing inducer concentrations. Results are mean values over 100 min.

After induction of the CRISPRi mechanism with aTc and IPTG, bacterial cells rapidly stopped division and started the expression of mVenus (which also was under the control of a pLTetO promoter). The cell length distribution of *ftsZ*-knockdown bacteria broadens and shifts towards greater lengths (Fig 3A). The mean cell length increases from <L> = 3 μm to <L> = 21 μm in three hours with 500 μM IPTG and 107 nM aTc (which we defined as the 100% induction level). We performed time-lapse video microscopy studies (cf. Materials and Methods) to observe the growth and fluorescence of individual filamentous bacteria over time. Microfluidic trap chambers were connected to fresh medium supply channels (S4 Fig). Upon 100% induction of the CRISPRi mechanism, we were able to switch to filamentous cell growth (with a division rate approaching zero, cf. inset of Fig 3B). As shown in Fig 3B the average length of the bacteria increased faster with higher inducer concentrations and continued exponential growth at 100% induction. A medium induction level (43%) results in a constant average cell length which is, however, larger than for normal growing and dividing cells. As found by Li *et al*. [25], at low induction levels there is a co-existence of subpopulations of normal growing and filamentous cells. Filamentous growth of *E*. *coli* proceeded up to 10 hours after which all bacteria had finally burst (S2 Movie).

### Efficiency of restoration of normal cell division

**Fig 4:**
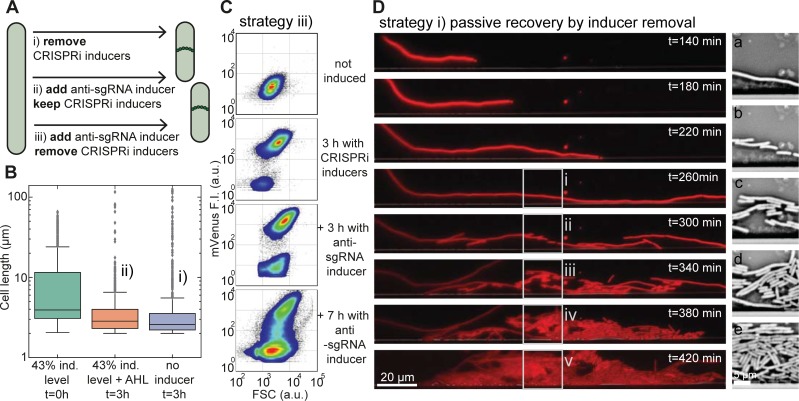
Restoration of cell division. (**A)** Scheme showing three different strategies by which filamentous bacteria can be returned to normal cell division and growth. (**B)** Box plot of cell lengths (1000 cells each) of induced filamentous cells (at t = 0 hours) and their length after 3 h in medium supplemented with AHL (in the presence of IPTG, aTc), or after 3 hours in medium without any inducers. For both rescue strategies, the mean cell size shifted back from <L> = 10 μm to <L> = 4 μm. **(C)** Flow cytometer density plots of bacteria displaying mVenus fluorescence vs. forward scatter (FSC) signal from a non-induced, normal growth state (top) towards the filamentous state by induction with IPTG/aTc (second) and back again (bottom) by supplementation of AHL (in the presence of IPTG/aTc). Both the mVenus and FSC signals increase in the filamentous growth mode. After the release of anti-sgRNA, the size of the filamentous population decreases and recovers a normal cell morphology. **(D)** Left column: Fluorescence microscopy time series in the mRFP channel of an *E*. *coli* cell in a microfluidic chamber in the absence of inducers. Prior to the first image (top), the bacterium was grown filamentous for two hours at 43% induction level followed by a 140-minute exposure to growth medium without any inducers. At 300 minutes of growth, the bacterium resumes cell division. The new daughter cells quickly approach the normal cell size. Right column: images (a)-(e) show bright field images corresponding to the boxed regions on the left.

We investigated three switching strategies to revert filamentous cells back to normal growth (Fig 4A): i) passive switching by stopping the production of dCas9-sgRNAs via removal of CRISPRi inducers aTc and IPTG (Fig 4D); ii) by induction of anti-sgRNAs in the presence of CRISPRi inducers (the ‘antisense’ strategy), or iii) by removal of CRISPRi inducers and addition of anti-sgRNA inducer (the ‘active’ approach). We investigated the three strategies using bulk measurements on liquid cultures and single cell data obtained from flow cytometry and time-lapse fluorescence microscopy using microfluidic cell traps.

In the bulk experiments, we found that the cell length distribution of a sample from a liquid culture after induction of CRISPRi had a mean of <L> = 10 μm, which shifted back to <L> = 4 μm after 3 hours using both the passive (i) and the antisense strategy (ii) (Fig 4B).

In case of the active strategy (iii) in the absence of CRISPRi inducers, we performed flow cytometry (FCM) experiments [29–31] to quantitatively monitor changes in cell morphology during the switching process. Here, the forward scatter (FSC) signal is roughly proportional to the cell size, while the side scatter (SSC) signal contains information about the cellular granularity [32, 33]. A change in cell morphology such as filamentation thus correlates with changes in FSC and SSC. Upon IPTG/aTc induction about 93% of the cells switch to higher mVenus (co-expressed with dCas9) fluorescence intensity and higher FSC signal (Fig 4C and S5 Fig). After supplementation of AHL (60 nM for 3 hours) the non-filamentous population already increased from 6% to 28%. The AHL concentrations used, however, did not strongly affect bacterial growth in corresponding control experiments (S6 Fig).

**Fig 5:**
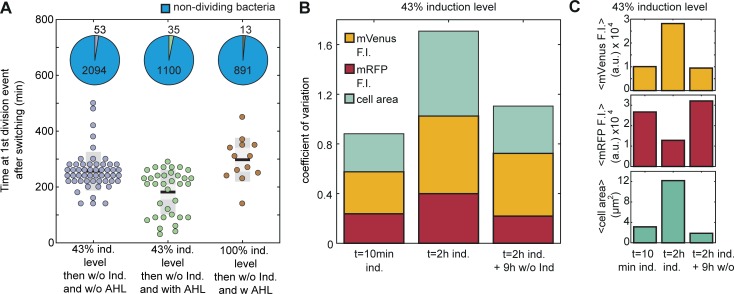
Single cell analysis of cell division switching. **(A)** The time delay between the removal of IPTG/aTc with and without AHL at the first cell division event for different inducer concentrations of IPTG/aTc. The pie charts show the fraction of cells that divided compared to the cells that did not divide for each condition. **(B)** The coefficient of variation was calculated for the fluorescence intensities of mVenus and mRFP and the cell area. The variability of the population increases during the 2 hours induction period and drops again for the new population emerging by switching, i.e., after removal of IPTG/aTc. **(C)** Upon aTc/IPTG induction, the cells shift towards higher mean mVenus fluorescence intensities, lower mean mRFP and higher mean cell area. After 9 hours without inducers the mean values return to the level of the first measurement (t = 10 min ind.).

All three strategies were investigated using microfluidics. To our surprise, in contrast to the bulk experiments we here usually did not observe re-initiation of cell division under strategy (ii), i.e., supplementation of AHL while aTc and IPTG are still present. From about 2000 analyzed cells only a single rescue event was observed for strategy (ii) as shown in S7 Fig. By contrast, the removal of the CRISPRi inducers–both with or without AHL–allowed a fraction of about 1.5–3% of all analyzed cells to resume normal cell division (Fig 5A and.S4 Movie). However, in the presence of AHL (iii) some cells started to divide again after filamentous growth significantly faster than in the absence of AHL (i) (Fig 5A). Furthermore, full induction of the CRISPRi compared to 43% induction level for about 2 hours delayed the timing of the first division event even in the presence of AHL.

Our experiments in microfluidic cell traps revealed a considerable phenotypic heterogeneity upon induction of filamentation, which was reversed once normal growth was restored (Fig 5B). In addition to their size, we characterized the cells with respect to their fluorescence intensity. After 2 hours of continuous induction with IPTG/aTc, the coefficient of variation (CV = standard deviation/mean) of the cell size as well as of mVenus and mRFP expression increased considerably. In contrast to aTc-induced mVenus (on the CRISPRi plasmid), the constitutively expressed mRFP (on the anti-sgRNA plasmid) level dropped during filamentation (Fig 5C). Additionally, most of the cells that divide within 120 minutes after the removal of the CRISPRi inducers with strategy (iii) have a mean mVenus fluorescence that is 0.2 times lower than the average of the main population (Fig 5A and S10 Fig).

## Discussion

In *E*. *coli* cells, a knockdown of the *ftsZ* gene stops cell division while still allowing cell growth, resulting in long filamentous cells. Restoration of FtsZ levels, in principle, allows cells to return back to normal growth. This opens up the possibility to implement a genetic switch that can turn cell division in *E*. *coli* off and on again. We here used a plasmid-based inducible CRISPRi system to reduce the FtsZ levels in *E*. *coli* and stop cell division. To implement the circuit in viable bacteria, decoy-binding sites for the FtsZ-suppressing dCas9-sgRNA complexes had to be introduced as genetic buffer elements. We then reverted the knockdown by removing the CRISPRi inducers and counter-acted CRISPRi utilizing inducible antisense-sgRNA molecules that could be used to adjust the threshold for bacterial filamentation and thus modulate the switching process. Due to the critical role of cell division in the bacterial life cycle, however, our CRISPRi-based approach strongly interfered with the physiology of the bacterial host chassis including slowing down cell division restoration in the majority of the cells.

We used microscopy time lapse experiments to follow thousands of cells through the switching process to understand the response to the genetic switch on the single cell level. We found that at best only about 1.5–3% of the analyzed cells revert back to normal growth whereas the rest of the cells lyse. However, expression of anti-sgRNAs can rescue cell division faster than in experiments, in which only the CRISPRi inducers are removed. Our experiments demonstrate both the possibilities and limitations of using dCas9 as a switchable repressor for genes such as *ftsZ* that affect bacterial fitness.

### Switching cell division off

The CRISPRi-mediated knockdown of *ftsZ* was a surprisingly efficient method to stop cell division. The microfluidic time-lapse videos of the filamentation process revealed that some bacterial cells can still undergo cell division, but the division rate is decreasing with the inducer concentration of the CRISPRi system resulting in a larger average cell length (Fig 3B). Furthermore, the distribution of cell lengths broadens with continuing CRISPRi induction (Fig 3A). We were able to identify at least three contributions to such a broadening distribution: filamentous cells do not necessarily divide symmetrically [34], larger cells can produce cell mass faster than smaller cells [35] and filamentous cells start to slow down their growth rate at some point and eventually lyse.

It was found that when the FtsZ level falls below ≈ 28% of its initial value, the cell is not able to form FtsZ rings [15]. We observed a reduction of the FtsZ level by about 80% (judged from RT-qPCR and Western Blotting), by targeting the template strand of the promoters (ftsZ2p, ftsZ3p, ftsZ4p) that, however, only account for about 14% of the total transcription of *ftsZ* in the cell [36]. Hence, a major fraction of the FtsZ level reduction (about 66%) may be caused by dCas9-sgRNA blocking the transcriptional elongation also from upstream promoters. It was previously found that targeting the non-template strand with the sgRNA results in stronger repression of transcriptional elongation [37]. Indeed, Elhadi *et al*., tested several sgRNA variants targeting different positions of the non-template strand within the *ftsZ* gene and found that by combining two different sgRNA variants the repression efficiency was above 95% [17].

In this context, dCas9 off-target kinetics have been previously studied [38]. Off-target binding strongly affects dissociation and association kinetics and thus occupancy of DNA sites, which could further contribute to gene expression heterogeneity. In addition, Cho *et al*. reported dCas9 binding to several genes even in the absence of guide RNA [27].

To our surprise, the plasmid-based inducible CRISPRi-system led to filamentous growth even in the absence of inducers (Panel A in S1 Fig). This stands in contrast to *ftsZ* knockdown using tunable CRISPRi (tCRISPRi) or other previously employed plasmid-based CRISPRi systems [17, 25]. One major difference is that we used an inducible T7 RNA Polymerase (RNAP) for the expression of the sgRNAs rather than constitutive *E*. *coli* promoters. We assume that the leaky expression of the T7 RNAP together with the leaky expression of dCas9 produces enough dCas9-sgRNA complexes to already sufficiently knock down *ftsZ* on the chromosome. Previously, strongly binding TFs such as TetR were buffered by the introduction of DNA sponges [39]. In a similar way, we here set the threshold for CRISPRi-based *ftsZ* knockdown via the sponge copy number, solving the problem of non-induced filamentation (Fig 1 and Panel B in S1 Fig). Thus, cellular filamentation in the present study could be clearly linked to the CRISPRi reduced FtsZ levels, which was supported by various control experiments. Potential CRISPRi off-target effects have not been studied in the context of this work, however.

### Restoration of *E*. *coli* to normal growth

Microfluidic time lapse videos showed that switching cell division back on was less efficient than anticipated from samples of the liquid bulk culture (Fig 4B) and from flow cytometry data (Fig 4C). Methods that lack an appropriate time resolution such as the two methods mentioned above, do not allow to distinguish between cells that have just switched back and daughter cells from already restored cells. This can lead to an overestimation of the switching efficiency, in particular when the new state of the bacteria is associated with increased fitness (which is the case here).

In all our switching strategies, restoration of normal growth for most cells occurred within a few hours, which is slower than in studies where *ftsZ* was directly put under the control of an IPTG-inducible promoter [15]. In our case, the removal of the inducers stops new dCas9-sgRNA complexes from being expressed and assembled, but does not influence already bound complexes. In particular, it has been shown that activated dCas9 binds to its recognized DNA target site very strongly (Kd ≈ 0.5 nM) [38, 40]. In general, transcription factors (TFs) with strong affinity for their DNA-binding site such as TetR do not unbind from their target site until an extremely low amount of only a few molecules per cell (corresponding to low nanomolar concentrations) is reached. In agreement with this consideration, Boyle *et al* found that dissociation of dCas9 from on-target sites with strong affinity can take many hours [38]. Recovery of FtsZ levels is thus expected to be governed by the dilution of the dCas9-sgRNA complexes, which is automatically provided by cell growth. Thus, cell division can resume at potential division sites as soon as FtsZ levels recover. Other studies aiming at the reversal of CRISPRi due to dilution, reported full restoration of expression levels of targeted fluorescent proteins after washing away CRISPRi inducers at around 350–480 min [16, 25]. Interestingly, with our approach, it took only about 260 minutes on average to observe the first division events after the removal of CRISPRi inducers, suggesting that cell division resumes even before the *ftsZ* levels are fully recovered. However, in microfluidic devices we also observe that the main part of the population does not survive the slow knockdown and recovery process (Fig 5A). Importantly, the expression of anti-sgRNA allows some cells to resume cell division faster than without anti-sgRNA (Fig 5A). As suggested by the results from the *in vitro* experiments (Fig 2 and S2 Fig), the anti-sgRNA inhibits complex formation of dCas9 and sgRNA by binding to the sgRNA. The partial recovery of mVenus fluorescence in the cell-free expression system by the addition of anti-sgRNA after repression by CRISPRi lets us assume that anti-sgRNA can even displace the sgRNA from the dCas9-sgRNA complex. Hence, the expression of anti-sgRNA in the cells probably reduces the amount of dCas9-sgRNA bound on DNA target sites by reducing the concentration of free dCas9-sgRNA complexes, and thus shifting the equilibrium between the bound and unbound state.

Another CRISPRi anti-sgRNA approach was recently conducted by Lee *et al*. [19]. The anti-sense RNA in this study was shown to fully recover the expression of a fluorescent reporter within 360 minutes, even in the presence of continuously expressed sgRNA. Furthermore, they were able to establish a relationship between the de-repression efficiency and the binding affinity of the anti-sgRNA to the sgRNA, supporting the hypothesis that de-repression by anti-sense RNA can be partially explained by equilibrium thermodynamics. In contrast to the simple strand invasion approach taken in our study, Lee *et al*. augmented their antisense-sgRNA with binding sites for the RNA chaperone Hfq, which apparently increased its de-repression efficiency.

Rather than increasing sgRNA-anti-sgRNA interactions in this manner, alternatively the binding strength of dCas9-sgRNA to its target may be weakened. For instance, Vigouroux *et al*. recently demonstrated that reduced complementarity between target DNA and sgRNA increases the probability of the dCas9-sgRNA complex being kicked off by RNA or DNA polymerase [41]. This could help in the release of kinetically trapped dCas9-sgRNA complexes on the DNA target site to the cytosol where they might be more accessible to anti-sgRNA.

### Heterogeneity in gene expression

After switching to filamentous growth, we observed an increase in population heterogeneity, which is reflected by altered reporter fluorescence levels, an increase in their coefficients of variation, and by varying bacterial length distributions (Fig 5B and S8–S10 Figs). The measured decrease in constitutively expressed mRFP fluorescence intensity could be caused by plasmid dilution, a reduced protein expression rate or both.

Although both plasmid copy number controls are based on a negative feedback mechanism that measures the concentration of the plasmids in the cells, it is unclear what happens to the copy number in filamentous cells [42] [43]. Furthermore, only the copy number of the sum of the pLysS and CRISPRi plasmid should remain fixed, allowing for larger ratios between the two. We assume that the subpopulation of bacteria that switches faster than the rest of the cells, and which can be characterized by low mVenus fluorescence (S10 Fig), has a low copy number of the CRISPRi plasmid. After switching back to normal cell growth, the new population of growing cells displayed CV values similar to the starting population.

## Materials and methods

### Plasmids

We constructed plasmids by Gibson assembly of synthetic DNA fragments into the target vector (pSB1K3 and CRISPRi plasmid 44249 from addgene). The final plasmid sequences can be found in S2 Table. The sender strain was constructed in an earlier study and contains the gene for LuxI synthase (BioBrick part BBa_C0261). The sgRNA, anti-sgRNA and sponge element sequences are listed in S1 Table.

### Bacterial cell culture

Experiments with filamentous cells were performed in *Escherichia coli* BL21 (DE3) pLysS. Although the pLysS plasmid has the same origin of replication as the CRISPRi plasmid we select with the antibiotics (Carbenicillin) for the CRISPRi plasmid to ensure its presence in the cells. Furthermore, we detect the inducible mVenus expression from the CRISPRi plasmid in the cells. Cells from glycerol stock were grown in 5 ml Luria-Bertani medium containing antibiotics selecting for both plasmids (CRISPRi and asgRNA plasmid) and incubated over night at 37°C and 250 rpm. The following day, cells were diluted 1:1000 and incubated for additional 4 h. Optical density (OD) values between 0.4–0.6 were obtained. From this batch, 1 ml of the culture was centrifuged and the pellet resuspended in 300 μl growth medium. The concentrated cells were immediately loaded on a microfluidic chamber until single or few bacteria were captured in the traps. In such a microfluidic chemostat with defined bacterial trap dimensions, we supplied the bacterial suspension constantly with fresh nutrients (LB medium, antibiotics and/or inducer chemicals or dyes) using a pressure flow controller (OB1, Elveflow).

### Fluorescence time-lapse microscopy

The microfluidic PDMS (Sylgard 182, Dow Corning) device was fabricated using standard soft lithography as previously described [44]. The microfluidic device is a combination of a gradient mixer [45] and bacterial traps designed with dimensions of 200 μm x 10–50 μm x 1 μm as a H-shaped chemostat (S1 Fig).

Time-lapse microscopy measurements were conducted on a Nikon Ti-Eclipse epi-fluorescence microscope controlled with NIS-Elements Imaging Software. The microscope was equipped with a sCMOS camera (Zyla, Andor), an automated x-y-stage (Prior Scientific, Cambridge, UK) and an incubator box (Okolab) to maintain an operation temperature of 37°C. All videos were recorded with 40x apochromatic magnification objectives. Every 5 to 20 min, images in phase contrast mode, YFP as well as RFP fluorescence mode (in combination with the appropriate filter sets) were taken for a total run time of up to 20 hours. The exposure times were automatically adjusted.

### Cell-free expression

For the cell-free assay, the sgRNA sequences were designed complementary to the non-template strand (sequences can be found in Supporting Information S3 Table). Anti-sgRNA and sgRNA where transcribed *in vitro* by T7 RNA Polymerase (NEB) from linear DNA (IDT) overnight and then extracted with Phenol-Chloroform. The concentration of the RNA was determined by comparing a SYBR Green II stained band in a denaturing PAGE (8M Urea at 45°C) to the RNA Ladder (NEB, N0364S). The plasmid with mVenus was purified using Phenol-Chloroform prior the reaction in the cell extract. The crude S30 cell extract was obtained by beat beating of a BL21- Rosetta2(DE3) mid-log phase culture with 0.1 mm glass beads in a Minilys device (Peqlab) and supplemented with an energy mix and reaction buffer as described in ref. [28]. Instead of 3-phosphoglyceric acid (3-PGA), phosphoenolpyruvate (PEP) was utilized as an energy source. dCas9 was His-tagged and purified by gravity- flow chromatography with Ni-NTA Agarose Beads (Qiagen). The fluorescence intensity was measured with a FLUOstar Omega plate reader (BMG) in 96-well plate (ibidi) at 37°C. The composition of a single cell-free reaction was: 33% (v/v) S30 cell extract mixed with 42% (v/v) buffer and 25% (v/v) DNA plus inducers. For the experiments with the PURE system (NEB E6800S), the linear DNA fragment coding for mVenus with a T7 RNAP promoter was amplified using a standard PCR reaction and purified with Monarch PCR & DNA Cleanup Kit (NEB T1030S).

### Flow cytometry

An overnight culture was diluted (1/100) to 5 ml culture in LB and supplemented with appropriate antibiotics. The cell suspension was incubated for 2 hours and then induced with 100 μM IPTG and 11 nM aTc. 1 ml cell suspension was centrifuged and the pellet was solved in 2 ml PBS. For measurements with a flow cytometer (Cube8, Partec), the sample was further diluted with PBS (1:3). The anti-sgRNA was induced with 60 nM AHL for 3 h and 7 h before measuring. Cultures were diluted every 3 hours to keep the bacteria in the exponential growth phase. For each experiment, 100.000–150.000 events were recorded in FSC, SSC and FL1 (488ex/536em) and FL2 (532ex/590em) mode for mVenus and RFP detection.

### RT-qPCR

For the relative quantification of FtsZ levels between normal growing, filamentous and rescued cells, an overnight culture was diluted 1:1000 in LB containing Carbenicillin and Kanamycin. After 5 h at 37°C, an OD value of 0.4 was reached and 1 ml of the cells was centrifuged at 4°C for 5 min at 5000 rcf. RNA isolation was performed according to the RNA isolation II protocol (Bioline) with the following exception for lysis: 2.5 μl (1.25U) Proteinase K (NEB) was added to the lysozyme-based step. After the addition of buffer RLY, the sample was flash frozen in liquid nitrogen and stored at -80°C until filamentous and switched back cell samples were collected.

The remaining suspension of normal growing cells was diluted to OD 0.1 and supplemented with inducers (1 mM IPTG, 107 nM aTc) in fresh medium for 2 h and collected as described above at OD 0.4. The filamentous cell sample was again diluted (1:100) and supplemented with all inducers (1 mM IPTG, 107 nM aTc and 100 nM AHL) in fresh medium for 3 h. After the cells reached OD 0.1 a second dilution step (1:100) was performed in fresh LB+inducers and the cells were grown overnight. Next day, the switched back cells were diluted (1:100) one more time into fresh medium with all inducers until OD 0.4 was reached and the cells were collected as described above, flash frozen in LN2 and kept at -80°C until the continuation of RNA isolation procedure.

The RNA yield and quality was analyzed with a NanoDrop absorbance spectrometer (Uninduced: 118ng/μl, A260/280 1.9; filamentous: 253 ng/μl, A260/280 2.09; switched back: 264 ng/μl A260/280 2.117). Since the uninduced RNA sample showed low A260/280 value, all samples were off-column DNAseI digested and repurifed on a silica column. The final values obtained for A260/280 were >2 for all samples. Isolated RNA was flash frozen in LN_2_ and stored at -80°C until reverse transcription was performed according to the Maxima H Minus First Strand Synthesis Kit and manual for RT-qPCR (ThermoFisher) with 500 ng RNA for each sample. The DNA sequences for the used gene specific primers can be found in the supporting information file (S4 Table). The cDNA samples were aliquoted and flash frozen in LN_2_ and kept at -80°C until RT-qPCR was performed.

The RT-qPCR reactions were performed on a BioRad IQ5 instrument with the following settings: dynamic well factor method by addition of 10 nM Fluorescein to each sample and detection in the FAM channel by cycling 1x 1 min 95°C, 45x 30 s 95°C -> 15 s 60°C, 1x melt curve 55–95°C. The reactions were prepared with 7 μl of 1:10 serial dilutions of the cDNA and LunaScript Universal MasterMix 2x (New England Biolabs) in white PCR stripes with flat lid (AB-1191, ThermoFisher). Three technical replicates were recorded for each sample (two for the minus reverse transcriptase control and one for the non-template control). The gene specific primers for *ftsZ* and the reference genes can be found in the supporting information file (S4 Table). The Cq values were determined by the instruments´ data analysis software for background corrected and fitted curves. The RT-qPCR raw data can be found in the Dryad data package (doi: 10.5061/dryad.t153690). Fold changes of gene expression were determined by the Paffl method [46].

### Western blotting

An overnight culture was diluted in LB to OD 0.25 and grown for three hours with and without inducers (107 nM aTc and 1 mM IPTG). The sample with inducers was split and diluted again (to OD 0.25) to create two samples, one with 107 nM aTc and 1 mM IPTG and the other with 107 nM aTc, 1 mM IPTG and 100 nM AHL. Before and after the dilution, 2 ml from each sample were pelleted and suspended in lysis buffer (50 mM Tris, 14 mM MgGlu, 60 mM KGlu, 1 mM DTT, 0.1% TritonX100, pH 7.7) so that each sample had a concentration of 5 μg/ml (calculated from the OD). The samples were lysed with sonication on ice, pelleted and the supernatant was denatured at 95°C in Lämmli buffer and resolved by sodium dodecyl sulfate-polyacrylamide gel electrophoresis. 20 μg of proteins were transferred to PVDF membranes using a semi-dry transfer-blot apparatus (Bio-Rad). The membranes were blocked with 5% (w/v) BSA in TBST overnight at 4°C and probed with anti-sera to FtsZ (1:1000, Agrisera) for 1,5 h at room temperature [47]. A TRITC anti-mouse secondary antibody (1:5000; Agrisera) was applied for 1 h at 4°C in 5% BSA in TBST for 1 h and the blot was imaged with a Typhoon FLA 9500 scanner (General Electric) in the Cy3 channel.

### Data analysis

Image analysis was performed using NIS-Elements (Nikon) and customized MATLAB software. Flow cytometry data were plotted with FlowJo.

## Supporting information

S1 Fig Filamentation control tests. First, we tested the decoy-binding site strategy under the non-induced state. The liquid culture bacteria contained the CRISPRi-plasmid without (A) and with (B) a high copy number sponge plasmid. Only with additional sponge elements, the cells grow and divide. In (C) we tested dCas9 influence on cell morphology. The cells with CRISPRi and anti-sgRNA plasmids grow normal under dCas9 induction (107 nM aTc) for 3 hours. The corresponding reporter gene mVenus is also expressed from a tet-promoter as well as constitutive mRFP. The image is an overlay of phase contrast, mRFP and mVenus fluorescent channels.
(PDF)

S2 Fig Cell-free control tests. We used mVenus under T7 promoter in the PURExpress cell-free system (New England Biolabs). The expression of mVenus (5 nM DNA template) is blocked by the supplementation of dCas9 (50 nM) (NEB) and sgRNA (100 nM) and is reactivated upon the addition of anti-sgRNA56 (1 μM). The data points are three technical replicates. Expression levels reached the micromolar range. The inset figure shows one replication experiment in our homemade cell-free system with transcription under T7 polymerase.
(PDF)

S3 Fig *ftsZ* levels in normal, filamentous and switched-back cells. (A) RT-qPCR data for target gene *ftsZ*. The fluorescence intensities of serial dilutions (1:10) of technical triplicates of non-induced samples and of 1:100 dilutions of induced (1 mM IPTG, 107 nM aTc) and switched back samples (1 mM IPTG, 107 nM aTc, 100 nM AHL) are shown in the plot. From non-induced serial dilution curves, the amplification efficiency of 110% (top inset figure) was calculated from the obtained slope of the linear fit of extracted Cq values of the standard curves (Cq values can be found in S5 Table). The melt curve (inset figure below) shows a single peak corresponding to a single amplification product. The shoulder in front of the peak belongs to the master mix containing low ROX dye. The data for a minus reverse transcriptase control (-RT) and a non-template control (NTC) are also plotted in the graph. (B) Immunoblot showing FtsZ levels of normal and filamentous (+aTc/IPTG) cells. Diluting and splitting the filamentous cell culture with and w/o AHL (in the presence of aTc and IPTG), shows FtsZ recovery with AHL.
(PDF)

S4 Fig Microfluidic trap dimensions for single cell measurements. (A) The medium inlet direction is indicated with arrow marks. The exchange of nutrients and waste products occurs via diffusion. The channel width and trap dimensions are given in the layout. (B) The cannel height is 15 μm whereas trap height is 1 μm. The traps are incorporated in a microfluidic gradient mixer [45].
(PDF)

S5 Fig Quantitation of the switching process using flow cytometry. The mRFP (top panel) and the SSC signal (bottom panel) are plotted against the FSC signal. mRFP is constitutively expressed in initially normal cells but also after induction of filamentation. After switching actively back with AHL in the presence of filamentation inducers IPTG/aTc for 3 h, a population with decreased mRFP signal arises, which finally represents the main population (after 7 h of asgRNA induction). In the filamentous state, the SSC signal ramps up in proportion with the FSC signal (t = 3 h with aTc/IPTG). However, rescued cells quickly recover and the majority of the cells turn back to the initial scatter plot position as measured with normal growing and dividing bacteria (cf. not induced and after t = 7 h with aTc/IPTG/AHL).
(PDF)

S6 Fig Cell growth and expression of fluorescent protein in bulk measurements. (A) Bacterial growth measured by monitoring the absorbance of the bacterial culture at λ = 600 nm for various inducer concentrations. Bacterial growth was not affected by the induction of filamentation with aTc and IPTG nor by the supplementation of AHL. Three samples for each induction level are shown. (B) Fluorescence intensity time traces of mVenus measured at λem = 540 nm (excitation at 540 nm). (C) Fluorescence intensity of mRFP measured at λem = 590 nm (excitation at 544 nm). The fluorescence signal obtained from mRFP was both delayed and reduced for samples with AHL.
(PDF)

S7 Fig Cell division in the presence of inducers IPTG/aTc/AHL. The images are an overlay of bright field (phase contrast) and the fluorescence channels of mVenus and mRFP. During the first 1 hour and 20 minutes the cells are exposed to 215 μM IPTG and 46 nM aTc (43%). After 1 hour, 60 nM of AHL is supplemented to the growth medium. One bacterium resumes cell division (marked by an arrow), which belongs to a persisting subpopulation. The time is shown in hours. Scale bar: 20 μm.
(PDF)

S8 Fig Passive switching in microfluidic chambers. 3D scatter plot with the mean mRFP and mVenus fluorescence levels of single bacteria plotted against the observed area of the corresponding cells for about 1000 cells at three different points in time: 10 minutes after induction (43% induction level), after 2 hours of continuous induction and after additional 9 hours without aTc/IPTG. About 5% of the filamentous cells divide (‘dividers’: black dots) again after the inducers have been removed from the growth medium.
(PDF)

S9 Fig Active switching in microfluidic chambers. Histogram of cell lengths. Actively switched cells (strategy iii) regain normal cell length distributions similar to t = 0 hours of starting induction of filamentous growth.
(PDF)

S10 Fig Cell division restoration in a subpopulation. (A) The subpopulation (yellow bars) is characterized by low mVenus (top) and medium mRFP (bottom) levels. (B) 3D scatter plot of an actively switched bacterial population (switching supportd by anti-sgRNA in strategy (iii). About 5–6% of the analyzed cells re-initiates division upon removal of IPTG/aTc from the growth medium while supplementing AHL (60 M) in order to induce the expression of anti-sgRNA (‘fast dividers’: black dots).
(PDF)

S1 Table Sequences of target sites, sponge elements, sgRNAs and anti-sgRNA. DNA sequences of the target sites of *ftsA* (Gene ID: 944778)) and the derived elements employed in this study.
(PDF)

S2 Table Plasmid sequences and description. The table shows the plasmid features of the constructed CRISPRi plasmid, the sponge plasmid and the anti-sgRNA plasmid in detail.
(PDF)

S3 Table Sequences for the cell-free assay. The DNA regions of interest in this study are summarized here.
(PDF)

S4 Table Sequences for RT-qPCR primers. RT-primers were used for cDNA synthesis and REV and FWD primer pairs were used in qPCR reactions. The amplification products were for ftsZ (gene ID 944786) 97 nucleotides, for *rrsB* (gene ID 948466) 158 nucleotides and for *cysG* (gene ID 947880) 105 nucleotides long.
(PDF)

S5 Table RT-qPCR statistics. Cq values for technical triplicates for reference genes *rrsB*, *cysG* and target gene *ftsZ* and their mean and standard deviation (StDiv) values.
(PDF)

S6 Table RT-qPCR amplification efficiency and goodness of the linear fit for *rrsB* and cysG. From the obtained Cq values (see S4 Table), the amplification efficiencies for reference genes *rrsB* and *cysG* were extracted from the linear fit equations.
(PDF)

S1 Movie This video shows E. coli (with the CRISPRi and anti-sgRNA plasmids) in a microfluidic chamber without inducers of the CRISPRI mechanism. The images are an overlay of BF/phase contrast and fluorescence channels of mVenus and mRFP. Time is shown as hh:mm …
(AVI)

S2 Movie This video shows filamentous growth of E. coli in microfluidic chambers upon induction with 500 μM IPTG and 107 nM aTc (100% level). The images are an overlay of BF/phase contrast and fluorescence channels of mVenus and mRFP. Time is shown as hh:mm …
(AVI)

S3 Movie Active switching in microfluidic chambers. Filamentous growth is induced (215 μM IPTG and 46 nM aTc) for 2 hours. From there on the freshly supplied medium does not contain IPTG and aTc, but is supplemented with 50 nM AHL. The video starts after 1 hour of induction. One cell starts to re-divide about 50 minutes after the medium change. The images are an overlay of BF/phase contrast and fluorescence channels of mVenus and mRFP. The time is shown in hh:mm.
(AVI)

S4 Movie Passive switching in microfluidic chambers. Filamentous growth is induced (215 μM IPTG and 46 nM aTc) for 2 hours. After the time window, the freshly supplied medium is without inducers. The images are an overlay of BF/phase contrast and fluorescence channels of mVenus and mRFP. This video shows a bacterium that has a relatively low growth rate during induction and takes relatively long to start re-division. The time is shown in hh:mm.
(AVI)
