# Supplementary figures and images for: Filamentation and restoration of normal growth in Escherichia coli using a combined CRISPRi sgRNA/antisense RNA approach
Source: PLoS One. 2018 Sep 11;13(9):e0198058. doi: 10.1371/journal.pone.0198058 (PMC6133276; doi:10.1371/journal.pone.0198058)

**A**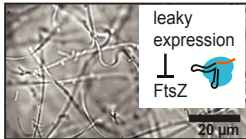**B**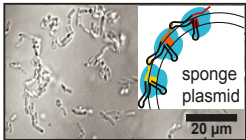**C**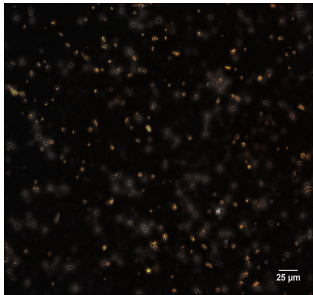

Supplement: S1 Fig — First, we tested the decoy-binding site strategy under the non-induced state. The liquid culture bacteria contained the CRISPRi-plasmid without (A) and with (B) a high copy number sponge plasmid. Only with additional sponge elements, the cells grow and divide. In (C) we tested dCas9 influence on cell morphology. The cells with CRISPRi and anti-sgRNA plasmids grow normal under dCas9 induction (107 nM aTc) for 3 hours. The corresponding reporter gene mVenus is also expressed from a tet-promoter as well as constitutive mRFP. The image is an overlay of phase contrast, mRFP and mVenus fluorescent channels. (PDF) [file pone.0198058.s001.pdf]

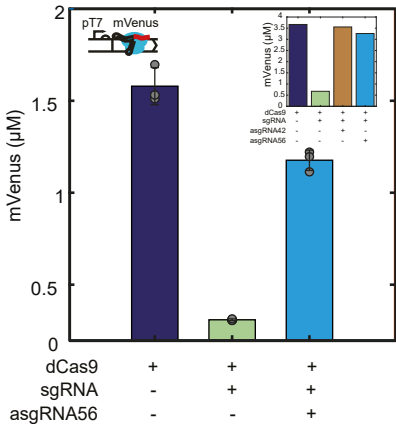

Supplement: S2 Fig — We used mVenus under T7 promoter in the PURExpress cell-free system (New England Biolabs). The expression of mVenus (5 nM DNA template) is blocked by the supplementation of dCas9 (50 nM) (NEB) and sgRNA (100 nM) and is reactivated upon the addition of anti-sgRNA56 (1 μM). The data points are three technical replicates. Expression levels reached the micromolar range. The inset figure shows one replication experiment in our homemade cell-free system with transcription under T7 polymerase. (PDF) [file pone.0198058.s002.pdf]

**A**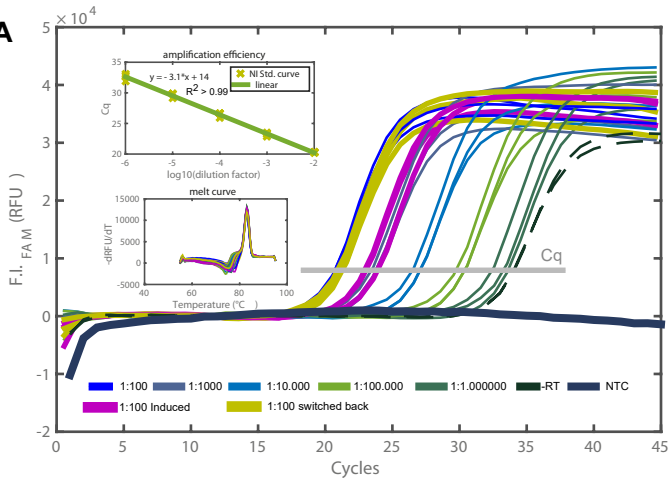**B**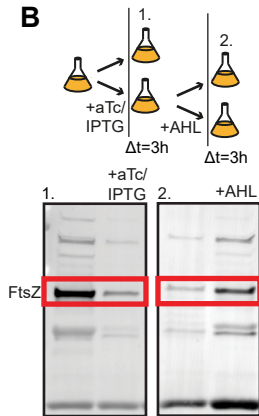

Supplement: S3 Fig — (A) RT-qPCR data for target gene ftsZ. The fluorescence intensities of serial dilutions (1:10) of technical triplicates of non-induced samples and of 1:100 dilutions of induced (1 mM IPTG, 107 nM aTc) and switched back samples (1 mM IPTG, 107 nM aTc, 100 nM AHL) are shown in the plot. From non-induced serial dilution curves, the amplification efficiency of 110% (top inset figure) was calculated from the obtained slope of the linear fit of extracted Cq values of the standard curves (Cq values can be found in S5 Table). The melt curve (inset figure below) shows a single peak corresponding to a single amplification product. The shoulder in front of the peak belongs to the master mix containing low ROX dye. The data for a minus reverse transcriptase control (-RT) and a non-template control (NTC) are also plotted in the graph. (B) Immunoblot showing FtsZ levels of normal and filamentous (+aTc/IPTG) cells. Diluting and splitting the filamentous cell culture with and w/o AHL (in the presence of aTc and IPTG), shows FtsZ recovery with AHL. (PDF) [file pone.0198058.s003.pdf]

**A**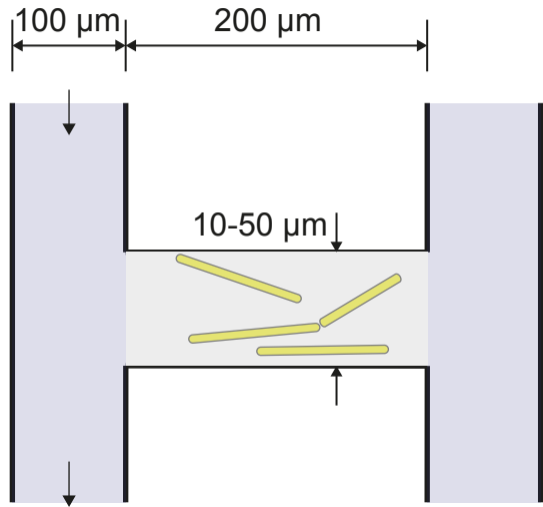**B**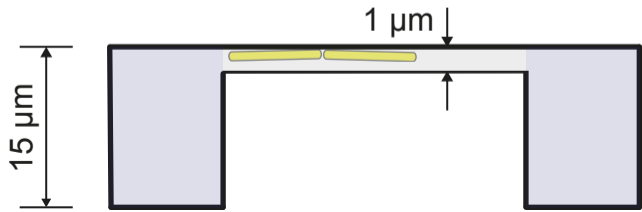

Supplement: S4 Fig — (A) The medium inlet direction is indicated with arrow marks. The exchange of nutrients and waste products occurs via diffusion. The channel width and trap dimensions are given in the layout. (B) The cannel height is 15 μm whereas trap height is 1 μm. The traps are incorporated in a microfluidic gradient mixer [45]. (PDF) [file pone.0198058.s004.pdf]

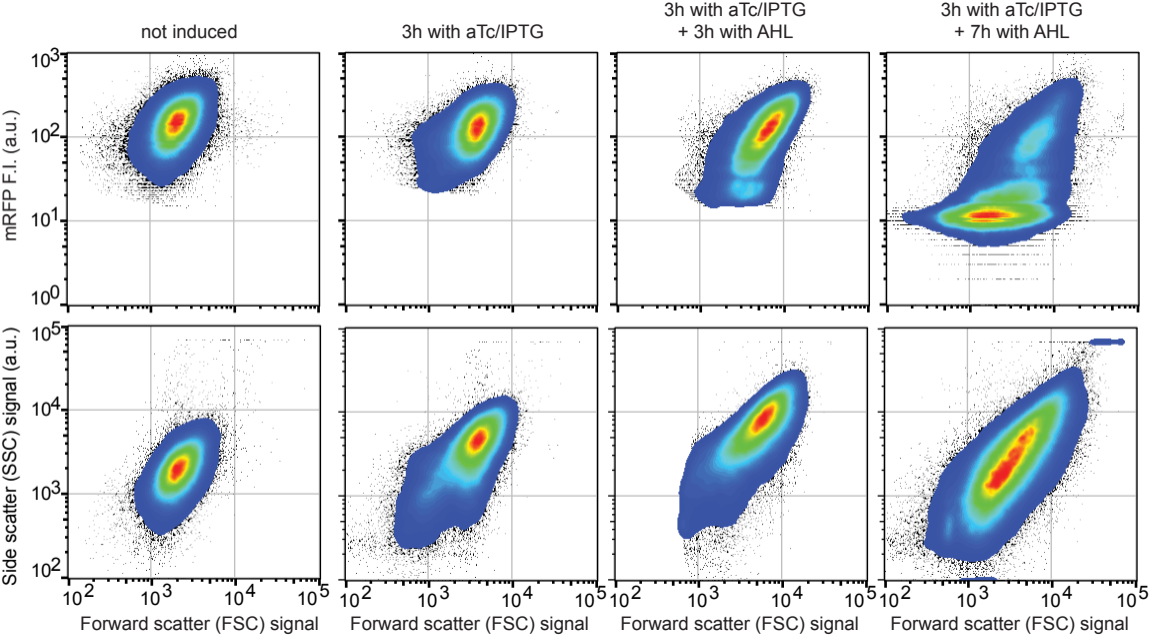

Supplement: S5 Fig — The mRFP (top panel) and the SSC signal (bottom panel) are plotted against the FSC signal. mRFP is constitutively expressed in initially normal cells but also after induction of filamentation. After switching actively back with AHL in the presence of filamentation inducers IPTG/aTc for 3 h, a population with decreased mRFP signal arises, which finally represents the main population (after 7 h of asgRNA induction). In the filamentous state, the SSC signal ramps up in proportion with the FSC signal (t = 3 h with aTc/IPTG). However, rescued cells quickly recover and the majority of the cells turn back to the initial scatter plot position as measured with normal growing and dividing bacteria (cf. not induced and after t = 7 h with aTc/IPTG/AHL). (PDF) [file pone.0198058.s005.pdf]

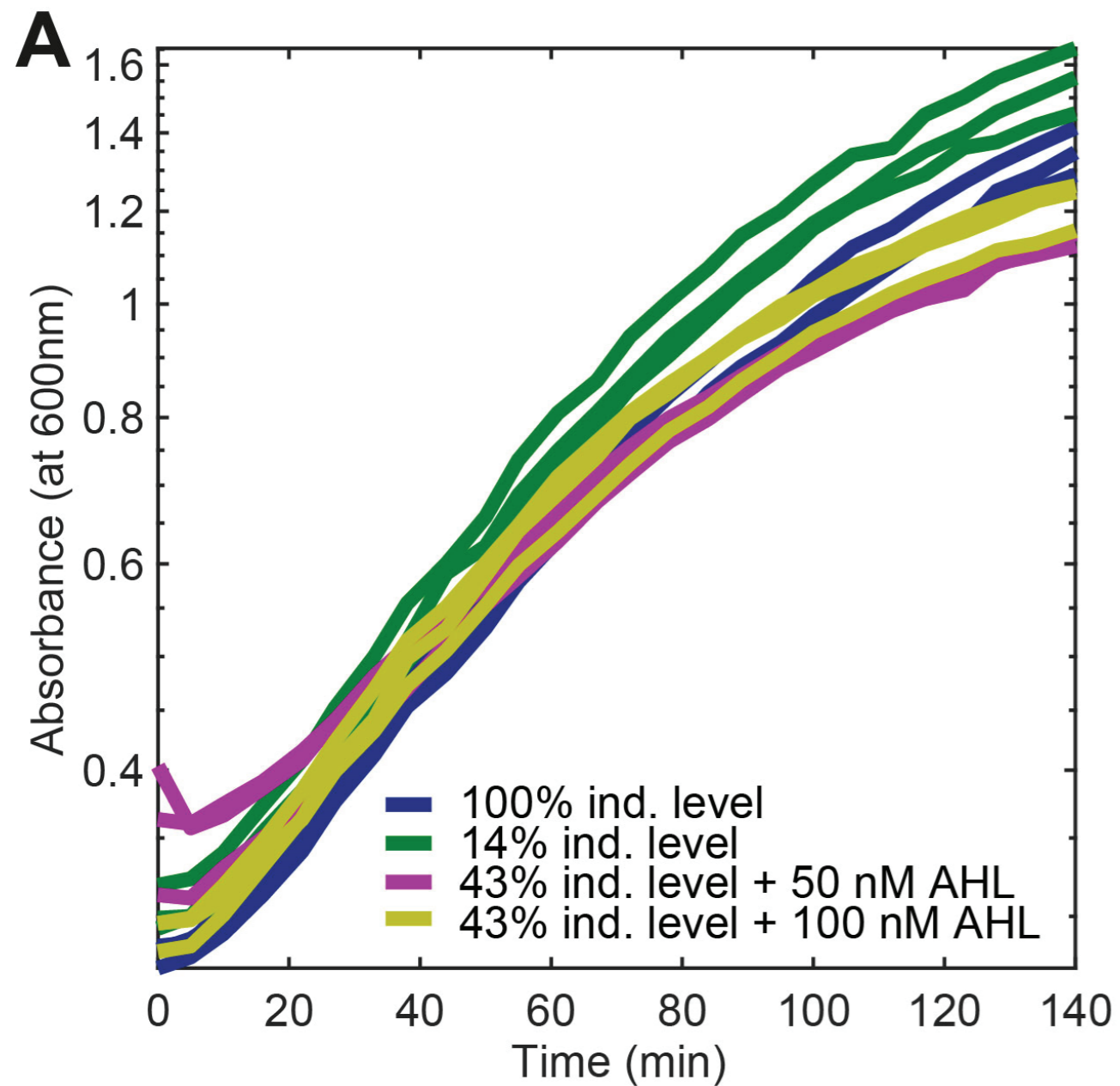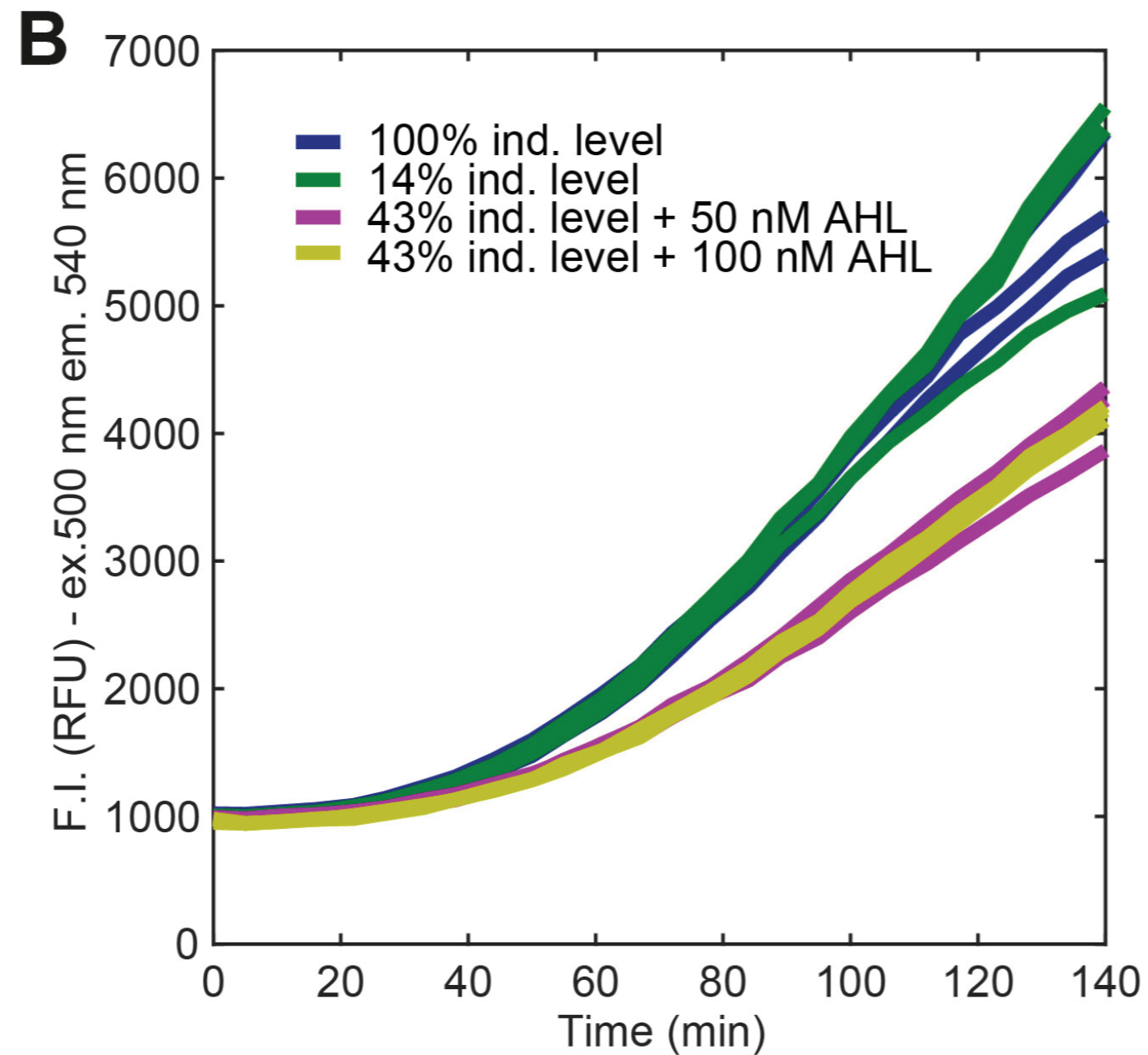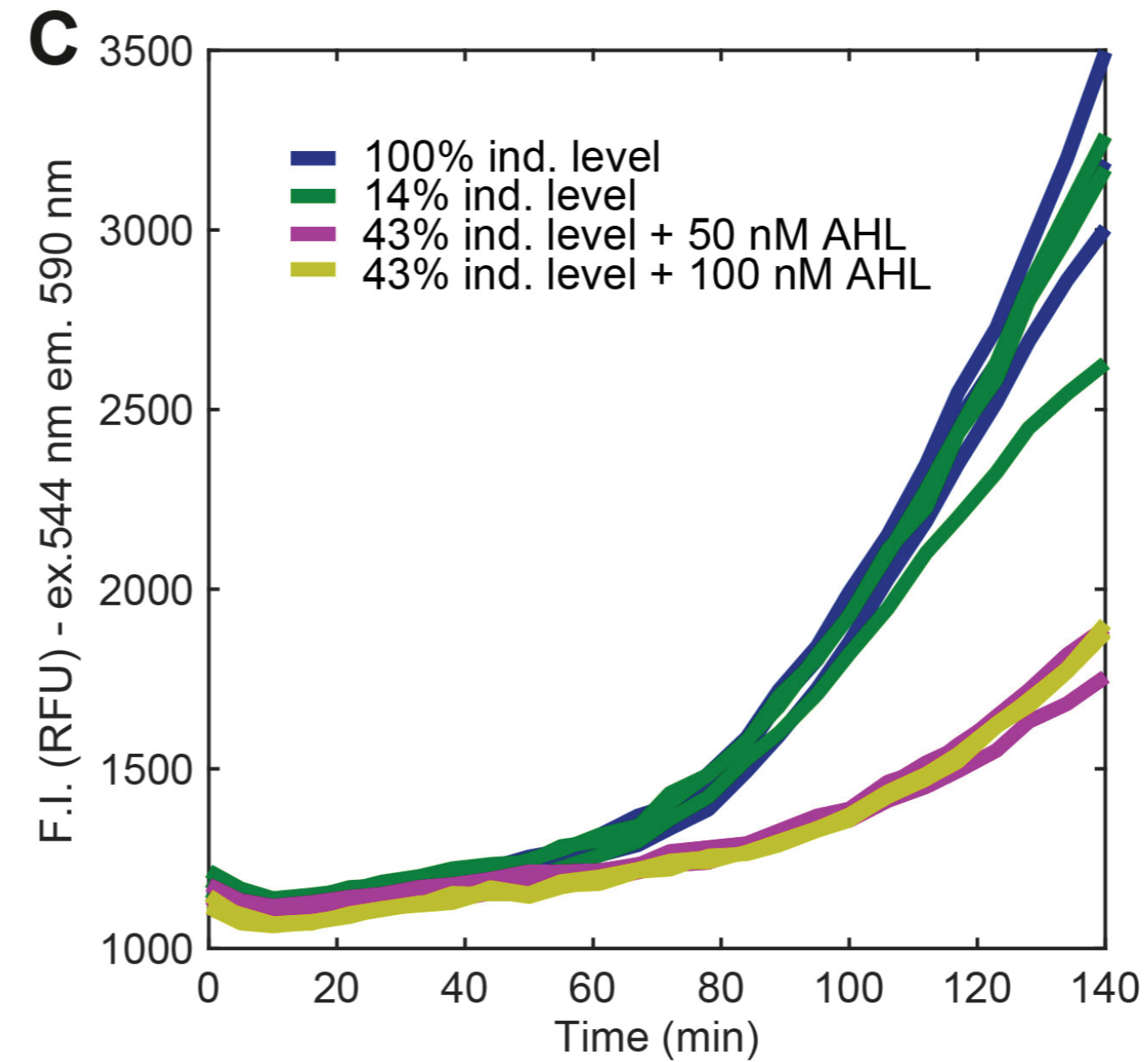

Supplement: S6 Fig — (A) Bacterial growth measured by monitoring the absorbance of the bacterial culture at λ = 600 nm for various inducer concentrations. Bacterial growth was not affected by the induction of filamentation with aTc and IPTG nor by the supplementation of AHL. Three samples for each induction level are shown. (B) Fluorescence intensity time traces of mVenus measured at λem = 540 nm (excitation at 540 nm). (C) Fluorescence intensity of mRFP measured at λem = 590 nm (excitation at 544 nm). The fluorescence signal obtained from mRFP was both delayed and reduced for samples with AHL. (PDF) [file pone.0198058.s006.pdf]

+IPTG/aTc

+IPTG/aTc  
+AHL

+IPTG/aTc  
+AHL

+IPTG/aTc  
+AHL

+IPTG/aTc  
+AHL

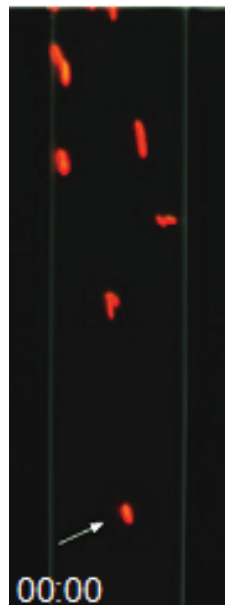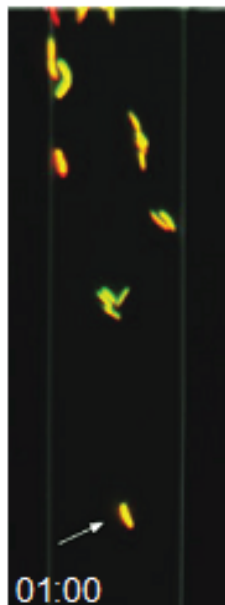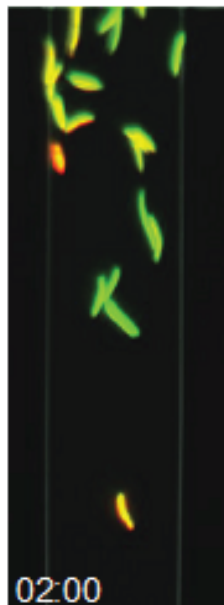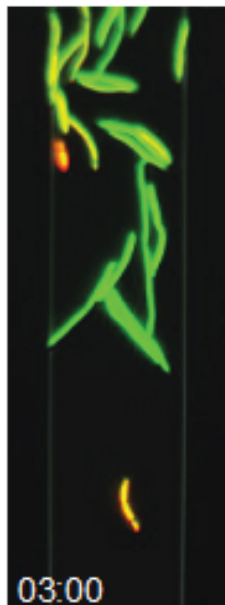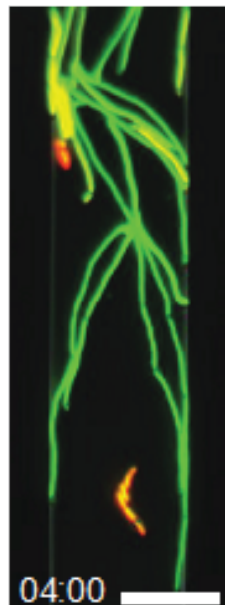

Supplement: S7 Fig — The images are an overlay of bright field (phase contrast) and the fluorescence channels of mVenus and mRFP. During the first 1 hour and 20 minutes the cells are exposed to 215 μM IPTG and 46 nM aTc (43%). After 1 hour, 60 nM of AHL is supplemented to the growth medium. One bacterium resumes cell division (marked by an arrow), which belongs to a persisting subpopulation. The time is shown in hours. Scale bar: 20 μm. (PDF) [file pone.0198058.s007.pdf]

**A**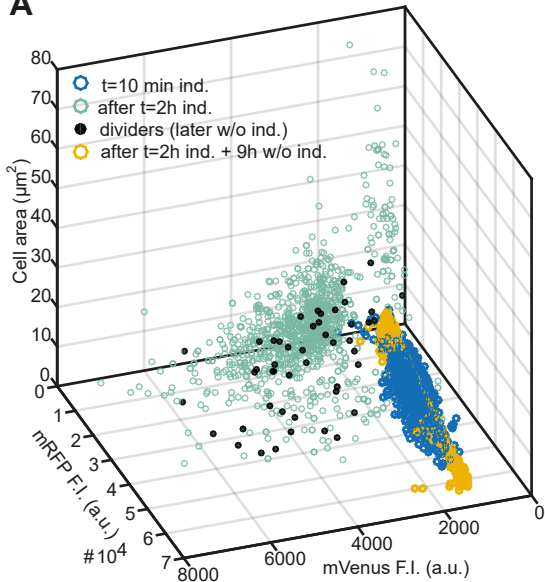

Supplement: S8 Fig — 3D scatter plot with the mean mRFP and mVenus fluorescence levels of single bacteria plotted against the observed area of the corresponding cells for about 1000 cells at three different points in time: 10 minutes after induction (43% induction level), after 2 hours of continuous induction and after additional 9 hours without aTc/IPTG. About 5% of the filamentous cells divide (‘dividers’: black dots) again after the inducers have been removed from the growth medium. (PDF) [file pone.0198058.s008.pdf]

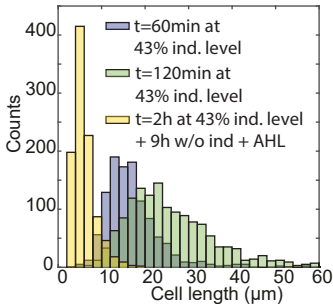

Supplement: S9 Fig — Histogram of cell lengths. Actively switched cells (strategy iii) regain normal cell length distributions similar to t = 0 hours of starting induction of filamentous growth. (PDF) [file pone.0198058.s009.pdf]

**A**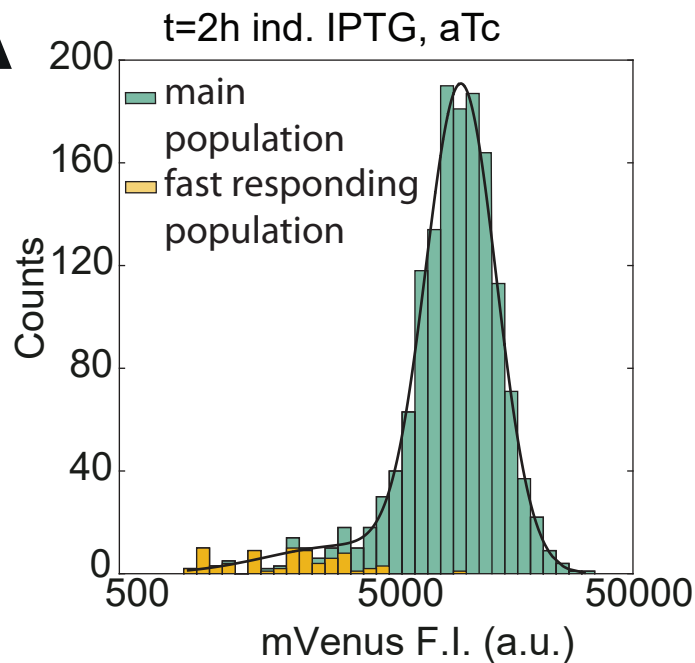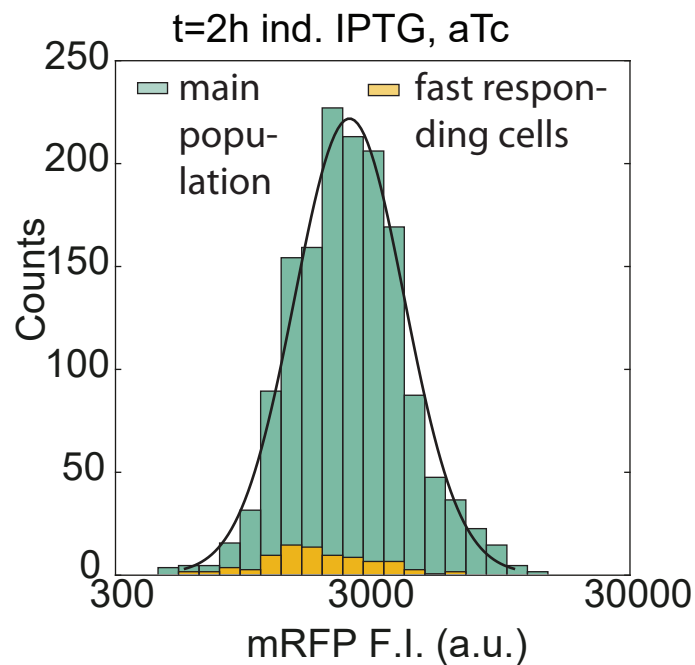**B**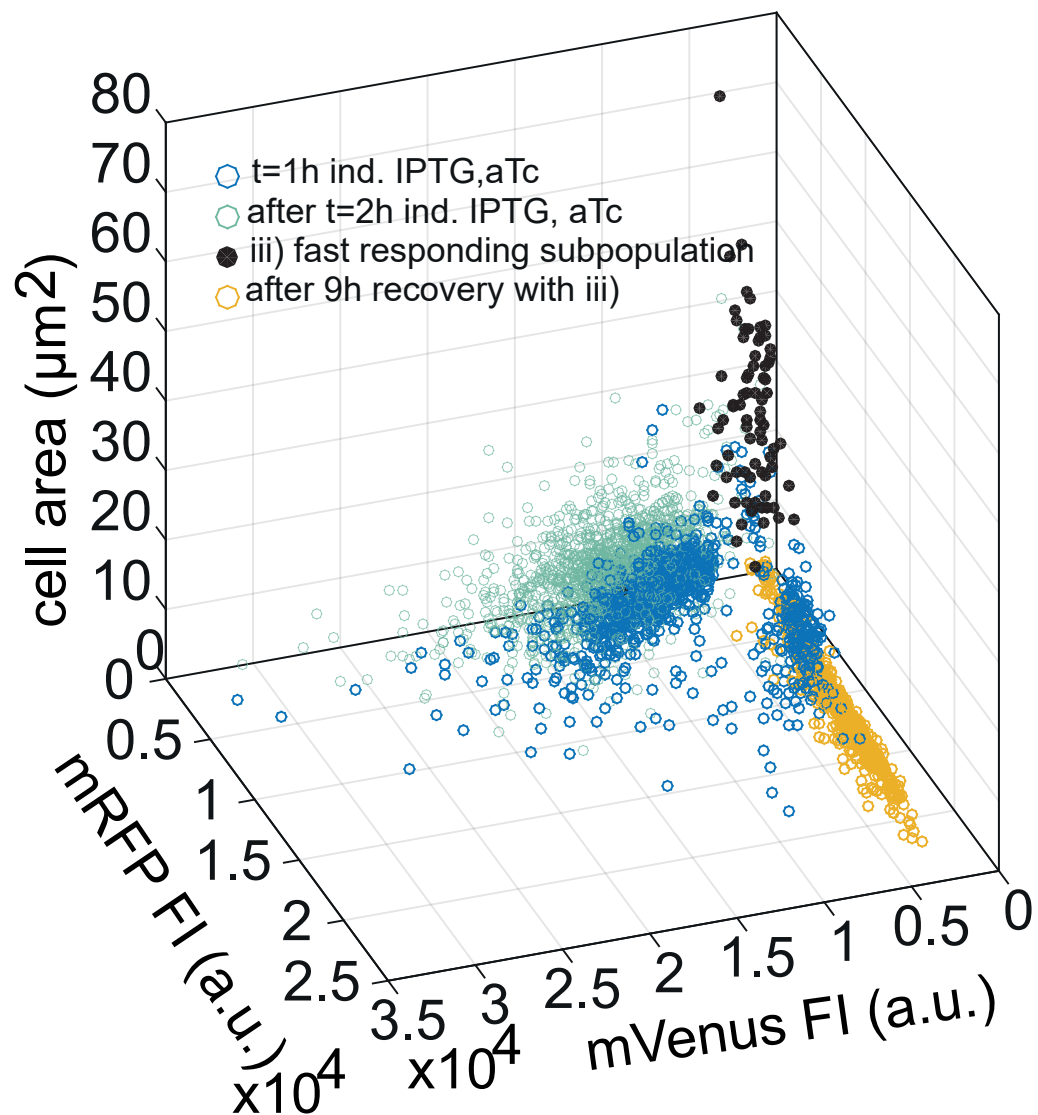

Supplement: S10 Fig — (A) The subpopulation (yellow bars) is characterized by low mVenus (top) and medium mRFP (bottom) levels. (B) 3D scatter plot of an actively switched bacterial population (switching supportd by anti-sgRNA in strategy (iii). About 5–6% of the analyzed cells re-initiates division upon removal of IPTG/aTc from the growth medium while supplementing AHL (60 M) in order to induce the expression of anti-sgRNA (‘fast dividers’: black dots). (PDF) [file pone.0198058.s010.pdf]
