# Supplementary material for: Filamentation and restoration of normal growth in Escherichia coli using a combined CRISPRi sgRNA/antisense RNA approach
Source: PLoS One. 2018 Sep 11;13(9):e0198058. doi: 10.1371/journal.pone.0198058 (PMC6133276; doi:10.1371/journal.pone.0198058)
Supplement: S1 Table — DNA sequences of the target sites of ftsA (Gene ID: 944778)) and the derived elements employed in this study. (PDF) [file pone.0198058.s011.pdf]

pZ2 target site and sponge element<br>tttcatacgcaagtgcgtat**cgg**
pZ3 target site and sponge element<br>actaaggtaattccttatgc**tgg**
pZ4 target site and sponge element<br>gtctgcgtcgtcgatatcgg**tgg**
pZ2 sgRNA<br>gggtttcatacgcaagtgcgtatgtttaagagctatgctggaaacagcatagcaagtttaaataaggctagtccgttatcaacttgaaaaagtggcaccgagtcggtgcttttttt
pZ3 sgRNA<br>gggactaaggtaattccttatgcgtttaagagctatgctggaaacagcatagcaagtttaaataaggctagtccgttatcaacttgaaaaagtggcaccgagtcggtgcttttttt
pZ4 sgRNA<br>ggggtctgcgtcgtcgatatcgggtttaagagctatgctggaaacagcatagcaagtttaaataaggctagtccgttatcaacttgaaaaagtggcaccgagtcggtgcttttttt
pZ3 anti-sgRNA<br>cactttttcaagttgataacggactagccttatttaaacttgctatgctgtttccagcatagctcttaaacgcataaggaattaccttagtccgcaaaaaaccccgcttcggcggggttttttcgc
pZ4-pZ2 anti-sgRNA<br>caagtaattgtcaacaaactccagcatagctcttaaacccgatatcgacgacgcagacttttttttttccagcatagctcttaaacatacgcacttgcgtatgaaacagagaatataaaaagccagattattaatccggcttttttattattt
