# Supplementary material for: Filamentation and restoration of normal growth in Escherichia coli using a combined CRISPRi sgRNA/antisense RNA approach
Source: PLoS One. 2018 Sep 11;13(9):e0198058. doi: 10.1371/journal.pone.0198058 (PMC6133276; doi:10.1371/journal.pone.0198058)
Supplement: S2 Table — The table shows the plasmid features of the constructed CRISPRi plasmid, the sponge plasmid and the anti-sgRNA plasmid in detail. (PDF) [file pone.0198058.s012.pdf]

The CRISPRi plasmid was purchased from addgene (#44249). Resistance was replaced to AmpR. dCas9 is under control of a tet promoter. The plasmid was modified with sponge elements, identical to the sequences of the FtsZ promoters pZ2-4 (2) (see Table S1). The sgRNAs for the pZ2-4 sites were transcribed from a T7 phage promoter. mVenus gene was cloned as reporter protein under the TetR repressible promoter (BBa:_R0040). The sequence is:

ATTCGCAAACGCCCTCTAATCGAAACTAATGGGGAAACTGGAGAAATTGTCTGGGATAAAGGGCGAGATTTTGCAC
AGTGCGCAAAGTATTGTCCATGCCCCAAGTCAATATTGTCAAGAAAACAGAAGTACAGACAGGCGGATTCTCCAAG
GAGTCAATTTTACCAAAAAGAAATTCGGACAAGCTTATTGCTCGTAAAAAAGACTGGGATCCAAAAAAATATGGTGG
TTTTGATAGTCCAACGGTAGCTTATTCAGTCCTAGTGGTTGCTAAGGTGGAAAAAGGGAAATCGAAGAAGTTAAAAT
CCGTTAAAGAGTTACTAGGGATCACAATTATGGAAAGAAGTTCCTTTGAAAAAAATCCGATTGACTTTTTAGAAGCTA
AAGGATATAAGGAAGTTAAAAAAGACTTAATCATTAAACTACCTAAATATAGTCTTTTTGAGTTAGAAAACGGTCGTA
AACGGATGCTGGCTAGTGCCGGAGAATTACAAAAAGGAAATGAGCTGGCTCTGCCAAGCAAATATGTGAATTTTTT
ATATTTAGCTAGTCATTATGAAAAGTTGAAGGGTAGTCCAGAAGATAACGAACAAAAACAATTGTTTGTGGAGCAGC
ATAAGCATTATTTAGATGAGATTATTGAGCAAATCAGTGAATTTTCTAAGCGTGTTATTTTAGCAGATGCCAATTTAG
ATAAAGTTCTTAGTGCATATAACAAACATAGAGACAAACCAATACGTGAACAAGCAGAAAATATTATTCATTTATTTAC
GTTGACGAATCTTGGAGCTCCCGCTGCTTTTAAATATTTTGATACAACAATTGATCGTAAACGATATACGTCTACAAA
AGAAGTTTTAGATGCCACTCTTATCCATCAATCCATCACTGGTCTTTATGAAACACGCATTGATTTGAGTCAGCTAGG
AGGTGACTAActcgagtaaggatctccaggcatcaaataaaacgaaaggctcagtcgaaagactgggcctttcgttttatctgttgtttgtcggtgaacgctctctact
agagtcacactggctcaccttcgggtgggcctttctgcgtttatacctagggatatattccgcttcctcgctcactgactcgctacgctcggtcgttcgactgcggcgagcgga
aatggcttacgaacggggcggagatttcctggaagatgccaggaagatacttaacagggaagtgagagggccgcggcaaagccgtttttccataggctccgcccccct
gacaagcatcacgaaatctgacgctcaaatcagtggtggcgaaacccgacaggactataaagataccaggcgtttccccctggcggctccctcgtgcgctctcctgttcc
tgcctttcggtttaccggtgtcattccgctgttatggccgcgtttgtctcattccacgcctgacactcagttccgggtaggcagttcgctccaagctggactgtatgcacgaaccc
cccgttcagtccgaccgctgcgccttatccggtaactatcgtcttgagtccaacccggaaagacatgcaaaagcaccactggcagcagccactggtaattgatttagagg
agttagtcttgaagtcatgcgccggttaaggctaaactgaaaggacaagttttggtgactgcgctcctccaagccagttacctcggttcaaagagttggtagctcagagaa
ccttcgaaaaaccgccctgcaaggcggttttttcgttttcagagcaagagattacgcgcagaccaaaacgatctcaagaagatcatcttattaatcagataaaatatttctag
atttcagtgcaatttatctcttcaaatgtagcacctgaagtcagccccatacgatataagttgttactagttcttacccgcaaaaaaccccgcttcggcgggtttttttcgcgctttt
aatttaCCAATGCTTAATCAGTGAGGCACCTATCTCAGCGATCTGTCTATTTCGTTCATCCATAGTTGCCTGACTCCCC
GTCGTGTAGATAACTACGATACGGGAGGGCTTACCATCTGGCCCCAGTGCTGCAATGATACCGCGAGACCCACGC
TCACCGGCTCCAGATTTATCAGCAATAAACCAGCCAGCCGGAAGGGCCGAGCGCAGAAGTGGTCCTGCAACTTTA
TCCGCCTCCATCCAGTCTATTAATTGTTGCCGGGAAGCTAGAGTAAGTAGTTCGCCAGTTAATAGTTTGCGCAACGT
TGTTGCCATTGCTACAGGCATCGTGGTGTCACGCTCGTCGTTTGGTATGGCTTCATTCAGCTCCGGTTCCCAACGA
TCAAGGCGAGTTACATGATCCCCCATGTTGTGCAAAAAAGCGGTTAGCTCCTTCGGTCCTCCGATCGTTGTCAGAA
GTAAGTTGGCCGCAGTGTTATCACTCATGGTTATGGCAGCACTGCATAATTCTCTTACTGTCATGCCATCCGTAAGA
TGCTTTTCTGTGACTGGTGAGTACTCAACCAAGTCATTCTGAGAATAGTGTATGCGGCGACCGAGTTGCTCTTGCC
CGGCGTCAATACGGGATAATACCGCGCCACATAGCAGAACTTTAAAAGTGCTCATcattggaaaacgttcttcggggcgaaaactc
tcaaggatcttaccgctgttgagatccagttcgatgtaacccactcgtgcacccaactgatcttcagcatcttttactttcaccagcgtttctgggtgagcaaaaacaggaagg
caaaatgccgcaaaaaagggaataagggcgacacggaaatgttgaatactcatactcttcctttttcaatcatgattgaagcatttatcagggttattgtctcatgagcggat
acatatttgaatgtatttagaaaaataaacaaattaattaataagccggcactaaggtaattccttatgctggatatatacgtgttccagttcttatcctatatcgaaagttcaatt
atacatgtctgcgtcgtcgatatcggtggcgtaccagtgtgttcgtgtcatctaggaggggcgcgtaggataaataatttttcatacgcaagtgcgtatgccggcatacgcac
ttgcgtatgaaaaattatttatatggtaatacgactcactatagggtttcatacgcaagtgcgtatgtttaagagctatgctggaaacagcatagcaagtttaaataaggctagt
ccgttatcaacttgaaaaagtggcaccgagtcggtgcttttttttaagtctctagactcgcacatgagcaaaacggtagagcgtcactttcactagtgtcgcggccgctaatac
gactcactatagggactaaggtaattccttatgcgtttaagagctatgctggaaacagcatagcaagtttaaataaggctagtccgttatcaacttgaaaaagtggcaccga
gtcggtgcttttttttaagtctctagagttgatccggattgactctatgacggtacctgctgactagtgcgcctgcaggatggtaatacgactcactatagggtctgcgtcgtcga
tatcgggtttaagagctatgctggaaacagcatagcaagtttaaataaggctagtccgttatcaacttgaaaaagtggcaccgagtcggtgcttttttttgcgctgcagccag
gcatcaaataaaacgaaaggctcagtcgaaagactgggcctttcgttttatctgttgtttgtcggtgaacgctctctactagagtcacactggctcaccttcgggtgggcctttc
tgcgtttatatttctagagtatggactagtgcttggattctcaccaataaaaaacgcccggcggcaaccgagcgttctgaacaaatccagatggagttctgaggtcattactg
gatctatcaacaggagtccaagcgagctcgatatcaaatccctatcagtgatagagattgacatccctatcagtgatagagatactgagcacgggtctatactagagaaa
gaggagaaatactagATGAGCAAAGGCGAAGAACTGTTCACGGGTGTGGTTCCGATCCTGGTTGAACTGGATGGCGATG
TGAACGGTCATAAATTTAGCGTGTCTGGTGAAGGCGAAGGTGATGCGACCTACGGCAAACTGACGCTGAAACTGAT
TTGCACCACGGGTAAACTGCCGGTTCCGTGGCCGACCCTGGTGACCACGCTGGGTTATGGTCTGATGTGTTTCGC
ACGTTACCCGGATCACATGAAACGCCATGATTTCTTTAAATCTGCGATGCCGGAAGGCTATGTGCAGGAACGTACC
ATCTTTTTCAAAGATGATGGTAACTACAAAACCCGCGCGGAAGTTAAATTTGAAGGCGATACGCTGGTGAACCGTAT
TGAACTGAAAGGTATCGATTTCAAAGAAGATGGCAATATTCTGGGTCACAAACTGGAATACAACTACAACAGTCATA
ACGTGTACATTACCGCCGATAAACAGAAAAACGGTATCAAAGCAAACTTCAAAATCCGTCACAACATCGAAGATGGC
GGTGTTCAGCTGGCCGATCATTACCAGCAGAACACCCCGATTGGCGATGGTCCGGTGCTGCTGCCGGATAATCAT
TATCTGAGTTACCAGAGCAAACTGTCTAAAGATCCGAATGAAAAACGCGATCACATGGTTCTGCTGGAATTTGTGAC
CGCGGCCGGCATTACGCATGGTATGGATGAACTGTATAAAtaacgtctcattttcgccagatatcgacgtcttaagacccactttcacatttaagt
tgtttttctaatccgcatatgatcaattcaaggccgaataagaaggctggctctgcaccttggtgatcaaataattcgatagcttgtcgtaataatggcggcatactatcagtag
taggtgtttccctttcttctttagcgacttgatgctcttgatcttccaatacgcaacctaaagtaaaatgccccacagcgctgagtgcatataatgcattctctagtgaaaaacctt
gttggcataaaaaggctaattgattttcgagagtttcatactgtttttctgtaggccgtgtacctaaatgtacttttgctccatcgcgatgacttagtaaagcacatctaaaacttta
gcgttattacgtaaaaaatcttgccagctttccccttctaaagggcaaaagtgagtatggtgcctatctaacatctcaatggctaaggcgtcgagcaaagcccgcttattttta
catgccaatacaatgtaggctgctctacacctagcttctgggcgagtttacgggttgttaaaccttcgattccgacctcattaagcagctctaatgcgctgttaatcactttactttt

atctaatctagacatcattaattcctaatttttgttgacactctatcgttgatagagttattttaccactccctatcagtgatagagaaaagaattcaaaagatctaaagaggaga
aaggatctATGGATAAGAAATACTCAATAGGCTTAGCTATCGGCACAAATAGCGTCGGATGGGCGGTGATCACTGATG
AATATAAGGTTCCGTCTAAAAAGTTCAAGGTTCTGGGAAATACAGACCGCCACAGTATCAAAAAAAATCTTATAGGG
GCTCTTTTATTTGACAGTGGAGAGACAGCGGAAGCGACTCGTCTCAAACGGACAGCTCGTAGAAGGTATACACGTC
GGAAGAATCGTATTTGTTATCTACAGGAGATTTTTTCAAATGAGATGGCGAAAGTAGATGATAGTTTCTTTCATCGAC
TTGAAGAGTCTTTTTTGGTGGAAGAAGACAAGAAGCATGAACGTCATCCTATTTTTGGAAATATAGTAGATGAAGTT
GCTTATCATGAGAAATATCCAACTATCTATCATCTGCGAAAAAAATTGGTAGATTCTACTGATAAAGCGGATTTGCGC
TTAATCTATTTGGCCTTAGCGCATATGATTAAGTTTCGTGGTCATTTTTTGATTGAGGGAGATTTAAATCCTGATAATA
GTGATGTGGACAAACTATTTATCCAGTTGGTACAAACCTACAATCAATTATTTGAAGAAAACCCTATTAACGCAAGTG
GAGTAGATGCTAAAGCGATTCTTTCTGCACGATTGAGTAAATCAAGACGATTAGAAAATCTCATTGCTCAGCTCCCC
GGTGAGAAGAAAAATGGCTTATTTGGGAATCTCATTGCTTTGTCATTGGGTTTGACCCCTAATTTTAAATCAAATTTT
GATTTGGCAGAAGATGCTAAATTACAGCTTTCAAAAGATACTTACGATGATGATTTAGATAATTTATTGGCGCAAATT
GGAGATCAATATGCTGATTTGTTTTTGGCAGCTAAGAATTTATCAGATGCTATTTTACTTTCAGATATCCTAAGAGTA
AATACTGAAATAACTAAGGCTCCCCTATCAGCTTCAATGATTAAACGCTACGATGAACATCATCAAGACTTGACTCTT
TTAAAAGCTTTAGTTCGACAACAACTTCCAGAAAAGTATAAAGAAATCTTTTTTGATCAATCAAAAAACGGATATGCA
GGTTATATTGATGGGGGAGCTAGCCAAGAAGAATTTTATAAATTTATCAAACCAATTTTAGAAAAAATGGATGGTACT
GAGGAATTATTGGTGAAACTAAATCGTGAAGATTTGCTGCGCAAGCAACGGACCTTTGACAACGGCTCTATTCCCC
ATCAAATTCACTTGGGTGAGCTGCATGCTATTTTGAGAAGACAAGAAGACTTTTATCCATTTTTAAAAGACAATCGTG
AGAAGATTGAAAAAATCTTGACTTTTCGAATTCCTTATTATGTTGGTCCATTGGCGCGTGGCAATAGTCGTTTTGCAT
GGATGACTCGGAAGTCTGAAGAAACAATTACCCCATGGAATTTTGAAGAAGTTGTCGATAAAGGTGCTTCAGCTCAA
TCATTTATTGAACGCATGACAAACTTTGATAAAAATCTTCCAAATGAAAAAGTACTACCAAAACATAGTTTGCTTTATG
AGTATTTTACGGTTTATAACGAATTGACAAAGGTCAAATATGTTACTGAAGGAATGCGAAAACCAGCATTTCTTTCAG
GTGAACAGAAGAAAGCCATTGTTGATTTACTCTTCAAAACAAATCGAAAAGTAACCGTTAAGCAATTAAAAGAAGATT
ATTTCAAAAAAATAGAATGTTTTGATAGTGTTGAAATTTCAGGAGTTGAAGATAGATTTAATGCTTCATTAGGTACCTA
CCATGATTTGCTAAAAATTATTAAAGATAAAGATTTTTTGGATAATGAAGAAAATGAAGATATCTTAGAGGATATTGTT
TTAACATTGACCTTATTTGAAGATAGGGAGATGATTGAGGAAAGACTTAAAACATATGCTCACCTCTTTGATGATAAG
GTGATGAAACAGCTTAAACGTCGCCGTTATACTGGTTGGGGACGTTTGTCTCGAAAATTGATTAATGGTATTAGGGA
TAAGCAATCTGGCAAAACAATATTAGATTTTTTGAAATCAGATGGTTTTGCCAATCGCAATTTTATGCAGCTGATCCA
TGATGATAGTTTGACATTTAAAGAAGACATTCAAAAAGCACAAGTGTCTGGACAAGGCGATAGTTTACATGAACATA
TTGCAAATTTAGCTGGTAGCCCTGCTATTAAAAAAGGTATTTTACAGACTGTAAAAGTTGTTGATGAATTGGTCAAAG
TAATGGGGCGGCATAAGCCAGAAAATATCGTTATTGAAATGGCACGTGAAAATCAGACAACTCAAAAGGGCCAGAA
AAATTCGCGAGAGCGTATGAAACGAATCGAAGAAGGTATCAAAGAATTAGGAAGTCAGATTCTTAAAGAGCATCCT
GTTGAAAATACTCAATTGCAAAATGAAAAGCTCTATCTCTATTATCTCCAAAATGGAAGAGACATGTATGTGGACCAA
GAATTAGATATTAATCGTTTAAGTGATTATGATGTCGATGCCATTGTTCCACAAAGTTTCCTTAAAGACGATTCAATA
GACAATAAGGTCTTAACGCGTTCTGATAAAAATCGTGGTAAATCGGATAACGTTCCAAGTGAAGAAGTAGTCAAAAA
GATGAAAAACTATTGGAGACAACTTCTAAACGCCAAGTTAATCACTCAACGTAAGTTTGATAATTTAACGAAAGCTGA
ACGTGGAGGTTTGAGTGAACTTGATAAAGCTGGTTTTATCAAACGCCAATTGGTTGAAACTCGCCAAATCACTAAGC
ATGTGGCACAAATTTTGGATAGTCGCATGAATACTAAATACGATGAAAATGATAAACTTATTCGAGAGGTTAAAGTGA
TTACCTTAAAATCTAAATTAGTTTCTGACTTCCGAAAAGATTTCCAATTCTATAAAGTACGTGAGATTAACAATTACCA
TCATGCCCATGATGCGTATCTAAATGCCGTCGTTGGAACTGCTTTGATTAAGAAATATCCAAAACTTGAATCGGAGT
TTGTCTATGGTGATTATAAAGTTTATGATGTTCGTAAAATGATTGCTAAGTCTGAGCAAGAAATAGGCAAAGCAACC
GCAAAATATTTCTTTTACTCTAATATCATGAACTTCTTCAAAACAGAAATTACACTTGCAAATGGAGAG

The high copy number sponge plasmid (backbone: pSB1K3) is compatible with the CRISPRi plasmid and was constructed with an *mrfp1* (BBa_J04450) reporter gene under control of a lac promoter We found that in LB medium this reporter is constitutively expressed. Furthermore, we added pZ2-4 sponge elements. The sequence is:

tactagtactaaggtaattccttatgctggatatatacgtgttccagttcttatcctatatcgaaagttcaattatacatgtctgcgtcgtcgatatcggtggcgtaccagtgtgttcg
tgtcatctaggaggggcgcgtaggataaataatttttcatacgcaagtgcgtatcggctgcagtccggcaaaaaagggcaaggtgtcaccaccctgcccttttctttaaaa
ccgaaaagattacttcgcgttatgcaggcttcctcgctcactgactcgctgcgctcggtcgttcggctgcggcgagcggtatcagctcactcaaaggcggtaatacggttat
ccacagaatcaggggataacgcaggaaagaacatgtgagcaaaaggccagcaaaaggccaggaaccgtaaaaaggccgcgttgctggcgtttttccacaggctcc
gcccccctgacgagcatcacaaaaatcgacgctcaagtcagaggtggcgaaacccgacaggactataaagataccaggcgtttccccctggaagctccctcgtgcgc
tctcctgttccgaccctgccgcttaccggatacctgtccgcctttctcccttcgggaagcgtggcgctttctcatagctcacgctgtaggtatctcagttcggtgtaggtcgttcgc
tccaagctgggctgtgtgcacgaaccccccgttcagcccgaccgctgcgccttatccggtaactatcgtcttgagtccaacccggtaagacacgacttatcgccactggca
gcagccactggtaacaggattagcagagcgaggtatgtaggcggtgctacagagttcttgaagtggtggcctaactacggctacactagaagaacagtatttggtatctg
cgctctgctgaagccagttaccttcggaaaaagagttggtagctcttgatccggcaaacaaaccaccgctggtagcggtggtttttttgtttgcaagcagcagattacgcgc
agaaaaaaaggatctcaagaagatcctttgatcttttctacggggtctgacgctcagtggaacgaaaactcacgttaagggattttggtcatgagattatcaaaaaggatct
tcacctagatccttttaaattaaaaatgaagttttaaatcaatctaaagtatatatgagtaaacttggtctgacagctcgagtcccgtcaagtcagcgtaatgctctgccagtgtt
acaaccaattaaccaattctgattagaaaaactcatcgagcatcaaatgaaactgcaatttattcatatcaggattatcaataccatatttttgaaaaagccgtttctgtaatga
aggagaaaactcaccgaggcagttccataggatggcaagatcctggtatcggtctgcgattccgactcgtccaacatcaatacaacctattaatttcccctcgtcaaaaat
aaggttatcaagtgagaaatcaccatgagtgacgactgaatccggtgagaatggcaaaagcttatgcatttctttccagacttgttcaacaggccagccattacgctcgtca
tcaaaatcactcgcatcaaccaaaccgttattcattcgtgattgcgcctgagcgagacgaaatacgcgatcgctgttaaaaggacaattacaaacaggaatcgaatgca
accggcgcaggaacactgccagcgcatcaacaatattttcacctgaatcaggatattcttctaatacctggaatgctgttttcccggggatcgcagtggtgagtaaccatgc
atcatcaggagtacggataaaatgcttgatggtcggaagaggcataaattccgtcagccagtttagtctgaccatctcatctgtaacatcattggcaacgctacctttgccat
gtttcagaaacaactctggcgcatcgggcttcccatacaatcgatagattgtcgcacctgattgcccgacattatcgcgagcccatttatacccatataaatcagcatccatg
ttggaatttaatcgcggcctggagcaagacgtttcccgttgaatatggctcataacaccccttgtattactgtttatgtaagcagacagttttattgttcatgatgatatatttttatctt
gtgcaatgtaacatcagagattttgagacacaacgtggctttgttgaataaatcgaacttttgctgagttgaaggatcagctcgagtgccacctgacgtctaagaaaccatta
ttatcatgacattaacctataaaaataggcgtatcacgaggcagaatttcagataaaaaaaatccttagctttcgctaaggatgatttctggaattcgcggccgcttctagag
caatacgcaaaccgcctctccccgcgcgttggccgattcattaatgcagctggcacgacaggtttcccgactggaaagcgggcagtgagcgcaacgcaattaatgtga
gttagctcactcattaggcaccccaggctttacactttatgcttccggctcgtatgttgtgtggaattgtgagcggataacaatttcacacatactagagaaagaggagaaat
actagATGGCTTCCTCCGAAGACGTTATCAAAGAGTTCATGCGTTTCAAAGTTCGTATGGAAGGTTCCGTTAACGGTC
ACGAGTTCGAAATCGAAGGTGAAGGTGAAGGTCGTCCGTACGAAGGTACCCAGACCGCTAAACTGAAAGTTACCA
AAGGTGGTCCGCTGCCGTTCGCTTGGGACATCCTGTCCCCGCAGTTCCAGTACGGTTCCAAAGCTTACGTTAAACA
CCCGGCTGACATCCCGGACTACCTGAAACTGTCCTTCCCGGAAGGTTTCAAATGGGAACGTGTTATGAACTTCGAA
GACGGTGGTGTTGTTACCGTTACCCAGGACTCCTCCCTGCAAGACGGTGAGTTCATCTACAAAGTTAAACTGCGTG
GTACCAACTTCCCGTCCGACGGTCCGGTTATGCAGAAAAAAACCATGGGTTGGGAAGCTTCCACCGAACGTATGTA
CCCGGAAGACGGTGCTCTGAAAGGTGAAATCAAAATGCGTCTGAAACTGAAAGACGGTGGTCACTACGACGCTGA
AGTTAAAACCACCTACATGGCTAAAAAACCGGTTCAGCTGCCGGGTGCTTACAAAACCGACATCAAACTGGACATC
ACCTCCCACAACGAAGACTACACCATCGTTGAACAGTACGAACGTGCTGAAGGTCGTCACTCCACCGGTGCTtaataa
cgctgatagtgctagtgtagatcgctactagagccaggcatcaaataaaacgaaaggctcagtcgaaagactgggcctttcgttttatctgttgtttgtcggtgaacgctctct
actagagtcacactggctcaccttcgggtgggcctttctgcgtttata

The anti-sgRNA elements (see Table S1) were cloned onto a high copy plasmid (backbone: pSB1K3) and are inducible with AHL since luxR (BBa_C0062) is expressed constitutively and they have a pLux promoter (BBa_R0062) The plasmid also carries sponge elements and is compatible with co-transformation with the CRISPRi plasmid. The sequence is:

tactagtactaaggtaattccttatgctggatatatacgtgttccagttcttatcctatatcgaaagttcaattatacatgtctgcgtcgtcgatatcggtggcgtaccagtgtgttcg
tgtcatctaggaggggcgcgtaggataaataatttttcatacgcaagtgcgtatcggctgcagtccggcaaaaagggcaaggtgtcaccaccctgcccttttttctttaaaa
ccgaaaagattacttcgcgttatgcaggcttcctcgctcactgactcgctgcgctcggtcgttcggctgcggcgagcggtatcagctcactcaaaggcggtaatacggttat
ccacagaatcaggggataacgcaggaaagaacatgtgagcaaaaggccagcaaaaggccaggaaccgtaaaaaggccgcgttgctggcgtttttccacaggctcc
gcccccctgacgagcatcacaaaaatcgacgctcaagtcagaggtggcgaaacccgacaggactataaagataccaggcgtttccccctggaagctccctcgtgcgc
tctcctgttccgaccctgccgcttaccggatacctgtccgcctttctcccttcgggaagcgtggcgctttctcatagctcacgctgtaggtatctcagttcggtgtaggtcgttcgc
tccaagctgggctgtgtgcacgaaccccccgttcagcccgaccgctgcgccttatccggtaactatcgtcttgagtccaacccggtaagacacgacttatcgccactggc
agcagccactggtaacaggattagcagagcgaggtatgtaggcggtgctacagagttcttgaagtggtggcctaactacggctacactagaagaacagtatttggtatct
gcgctctgctgaagccagttaccttcggaaaaagagttggtagctcttgatccggcaaacaaaccaccgctggtagcggtggtttttttgtttgcaagcagcagattacgcg
cagaaaaaaaggatctcaagaagatcctttgatcttttctacggggtctgacgctcagtggaacgaaaactcacgttaagggattttggtcatgagattatcaaaaaggat
cttcacctagatccttttaaattaaaaatgaagttttaaatcaatctaaagtatatatgagtaaacttggtctgacagctcgagtcccgtcaagtcagcgtaatgctctgccagt
gttacaaccaattaaccaattctgattagaaaaactcatcgagcatcaaatgaaactgcaatttattcatatcaggattatcaataccatatttttgaaaaagccgtttctgtaat
gaaggagaaaactcaccgaggcagttccataggatggcaagatcctggtatcggtctgcgattccgactcgtccaacatcaatacaacctattaatttcccctcgtcaaa
aataaggttatcaagtgagaaatcaccatgagtgacgactgaatccggtgagaatggcaaaagcttatgcatttctttccagacttgttcaacaggccagccattacgctc
gtcatcaaaatcactcgcatcaaccaaaccgttattcattcgtgattgcgcctgagcgagacgaaatacgcgatcgctgttaaaaggacaattacaaacaggaatcgaat
gcaaccggcgcaggaacactgccagcgcatcaacaatattttcacctgaatcaggatattcttctaatacctggaatgctgttttcccggggatcgcagtggtgagtaacc
atgcatcatcaggagtacggataaaatgcttgatggtcggaagaggcataaattccgtcagccagtttagtctgaccatctcatctgtaacatcattggcaacgctacctttg
ccatgtttcagaaacaactctggcgcatcgggcttcccatacaatcgatagattgtcgcacctgattgcccgacattatcgcgagcccatttatacccatataaatcagcatc
catgttggaatttaatcgcggcctggagcaagacgtttcccgttgaatatggctcataacaccccttgtattactgtttatgtaagcagacagttttattgttcatgatgatatattttt
atcttgtgcaatgtaacatcagagattttgagacacaacgtggctttgttgaataaatcgaacttttgctgagttgaaggatcagctcgagtgccacctgacgtctaagaaac
cattattatcatgacattaacctataaaaataggcgtatcacgaggcagaatttcagataaaaaaaatccttagctttcgctaaggatgatttctggaattcataaaaaaacgc
ccggcggcaaccgagcgttacctgtaggatcgtacaggtttacgcaagaaaatggtttgttatagtcgaataaacactttttcaagttgataacgacttgctatgctgtttcca
gcatagctcttaaacgcataaggaattaccttagtccgcaaaaaaccccgcttcggcggggtttttttcgccgacggtgtaggcacgtaagaggttccaactttcaccataat
gaaacagggtctatactagagaaagaggagaaatactagATGAAGAACATCAACGCGGATGATACGTACCGTATTATCAACAAGATT
AAAGCCTGCCGTTCGAACAACGATATCAACCAGTGCTTATCCGACATGACCAAAATGGTACATTGTGAGTACTACCT
GCTGGCGATCATTTATCCGCATAGCATGGTAAAATCAGATATTAGCATTCTCGACAACTATCCCAAGAAATGGCGTC
AGTATTATGATGACGCAAATCTCATCAAGTACGATCCGATCGTAGATTATTCTAACTCCAATCATTCACCCATTAACT
GGAATATCTTTGAAAACAACGCTGTTAACAAAAAAAGTCCCAATGTCATTAAAGAGGCCAAAACCTCGGGCCTGATT
ACCGGATTTTCCTTTCCAATCCATACGGCGAATAACGGCTTTGGCATGCTCTCTTTCGCACACAGTGAGAAGGACA
ACTATATTGATTCCCTTTTTCTTCATGCGTGCATGAATATTCCCCTGATCGTCCCGTCTCTGGTGGACAATTACCGC
AAGATCAATATTGCTAATAACAAAAGTAATAACGACCTTACGAAACGCGAGAAAGAGTGCCTGGCGTGGGCGTGCG
AAGGGAAAAGTTCGTGGGATATCTCTAAGATTCTTGGCTGTTCGGAGCGCACTGTGACCTTCCATCTGACGAACGC
CCAAATGAAATTAAACACGACGAATCGTTGCCAGTCCATCTCCAAAGCCATTCTGACGGGCGCAATCGACTGTCCG
TATTTTAAAAATTAAccaggcatcaaataaaacgaaaggctcagtcgaaagactgggcctttcgttttatctgttgtttgtcggtgaacgctctctactagagtcaca
ctggctcaccttcgggtgggcctttctgcgtttatataagtagggtcgaccaagaaccgcaagatgacctgtaggatcgtacaggtttacgcaagaaaatggtttgttatagt
cgaataaacaagtaattgtcaacaaactccagcatagctcttaaacccgatatcgacgacgcagactttttttttccagcatagctcttaaacatacgcacttgcgtatgaa
acagagaatataaaaagccagattattaatccggctttttattatttttccagatggagttctgaggtcattactggatctagagcaatacgcaaaccgcctctccccgcgcgtt
ggccgattcattaatgcagctggcacgacaggtttcccgactggaaagcgggcagtgagcgcaacgcaattaatgtgagttagctcactcattaggcaccccaggcttta
cactttatgcttccggctcgtatgttgtgtggaattgtgagcggataacaatttcacacatactagagaaagaggagaaatactagATGGCTTCCTCCGAAGAC
GTTATCAAAGAGTTCATGCGTTTCAAAGTTCGTATGGAAGGTTCCGTTAACGGTCACGAGTTCGAAATCGAAGGTG
AAGGTGAAGGTCGTCCGTACGAAGGTACCCAGACCGCTAAACTGAAAGTTACCAAAGGTGGTCCGCTGCCGTTCG
CTTGGGACATCCTGTCCCCGCAGTTCCAGTACGGTTCCAAAGCTTACGTTAAACACCCGGCTGACATCCCGGACTA
CCTGAAACTGTCCTTCCCGGAAGGTTTCAAATGGGAACGTGTTATGAACTTCGAAGACGGTGGTGTTGTTACCGTT
ACCCAGGACTCCTCCCTGCAAGACGGTGAGTTCATCTACAAAGTTAAACTGCGTGGTACCAACTTCCCGTCCGACG
GTCCGGTTATGCAGAAAAAAACCATGGGTTGGGAAGCTTCCACCGAACGTATGTACCCGGAAGACGGTGCTCTGA
AAGGTGAAATCAAAATGCGTCTGAAACTGAAAGACGGTGGTCACTACGACGCTGAAGTTAAAACCACCTACATGGC
TAAAAAACCGGTTCAGCTGCCGGGTGCTTACAAAACCGACATCAAACTGGACATCACCTCCCACAACGAAGACTAC
ACCATCGTTGAACAGTACGAACGTGCTGAAGGTCGTCACTCCACCGGTGCTtaataacgctgatagtgctagtgtagatcgctactag
agccaggcatcaaataaaacgaaaggctcagtcgaaagactgggcctttcgttttatctgttgtttgtcggtgaacgctctctactagagtcacactggctcaccttcgggtg
ggcctttctgcgtttata
