# Supplementary material for: Filamentation and restoration of normal growth in Escherichia coli using a combined CRISPRi sgRNA/antisense RNA approach
Source: PLoS One. 2018 Sep 11;13(9):e0198058. doi: 10.1371/journal.pone.0198058 (PMC6133276; doi:10.1371/journal.pone.0198058)
Supplement: S3 Table — The DNA regions of interest in this study are summarized here. (PDF) [file pone.0198058.s013.pdf]

mVenus zoom in into the DNA region of interest<br>…cacagtaatacgactcactatagggaattgtgagcggataacaattcccctctagaaataattttgtttaactttaagaaggagatatacatATGAGCAAAGGC<br>GAAGAACTGTTCACGGGTGTGGTT**CCG**ATCCTGGTTGAACTGGATGGCGATGTGA…
mVenus-sgRNA<br>GGGCCAUCCAGUUCAACCAGGAUGUUUAAGAGCUAUGCUGGAAACAGCAUAGCAAGUUUAAAUAAGGCUAGUCC<br>GUUAUCAACUUGAAAAAGUGGCACCGAGUCGGUCGUUUUUUUACACUGUCUGCAGUCCGGACACGCACC
mVenus anti-sgRNA(42)<br>GGGAUUUCCAGCAUAGCUCUUAAACAUCCUGGUUGAACUGGAUGGCCACACUGUCUGCAGUCCGGACACGCAC<br>CAG
mVenus anti-sgRNA(56)<br>GGGCACUUUUUCAAGUUGAUAACGACUUGCUAUGCUGUUUCCAGCAUAGCUCUUAAACAUCCUGGUUGAACUG<br>GAUGGCCACACUGUCUGCAGUCCGGACACGCACC

T7pol promoter and transcription start (GGG), pLacO, target site, dCas9handle, artificial terminator, **bold**: PAM site
