# Supplementary material for: Filamentation and restoration of normal growth in Escherichia coli using a combined CRISPRi sgRNA/antisense RNA approach
Source: PLoS One. 2018 Sep 11;13(9):e0198058. doi: 10.1371/journal.pone.0198058 (PMC6133276; doi:10.1371/journal.pone.0198058)
Supplement: S4 Table — RT-primers were used for cDNA synthesis and REV and FWD primer pairs were used in qPCR reactions. The amplification products were for ftsZ (gene ID 944786) 97 nucleotides, for rrsB (gene ID 948466) 158 nucleotides and for cysG (gene ID 947880) 105 nucleotides long. (PDF) [file pone.0198058.s014.pdf]

Reference genes	Target gene
**R_RT_cysG**	**R_RT_ftsZ**
CAGGTGTCGAGAAGGCTTTC	CAGTTTGTCGTTCGGGATAGT
**cysG_qPCR_FWD**	**ftsZ _qPCR_FWD**
TTGTCGGCGGTGGTGATGTC	CTTTGAAGGCAAGAAGCGTATG
**cysG_qPCR_REV**	**ftsZ _qPCR_REV**
ATGCGGTGAACTGTGGAATAAACG	CAGTTTGTCGTTCGGGATAGT
**R_RT_rrsB**	
GAGTCTGGACCGTGTCTCAG	
**rrsB_qPCR_FWD**	
GCTTGCTTCTTTGCTGACGAG	
**rrsB_qPCR_REV**	
ATCTGGGCACATCCGATGGC	
