# Supplementary material for: Filamentation and restoration of normal growth in Escherichia coli using a combined CRISPRi sgRNA/antisense RNA approach
Source: PLoS One. 2018 Sep 11;13(9):e0198058. doi: 10.1371/journal.pone.0198058 (PMC6133276; doi:10.1371/journal.pone.0198058)
Supplement: S5 Table — Cq values for technical triplicates for reference genes rrsB, cysG and target gene ftsZ and their mean and standard deviation (StDiv) values. (PDF) [file pone.0198058.s015.pdf]

rrsB	Cq	Cq_mean	StDev_Cq	*cysG*	Cq	Cq_mean	StDev_Cq	*ftsZ*	Cq	Cq_mean	StDev_Cq
1:100	8.1652	8.18	0.07	1:100	22.68	22.85	0.17	1:100	20.265	**20.27**	**0.10**
1:1000	12.967	12.01	0.89	1:1000	25.738	26.28	0.50	1:1000	23.268	23.22	0.30
1:10.000	16.357	15.96	0.38	1:10.000	28.988	29.36	0.33	1:10.000	26.618	26.39	0.41
1:100.000	19.297	19.02	0.25	1:100.000	32.633	32.20	0.38	1:100.000	29.87	29.63	0.41
1:1.000000	22.607	22.34	0.32	I_1:100	22.927	23.04	0.10	1:1.000000	33.111	32.57	0.64
I_1:100	8.5986	7.92	0.58	SwB_1:100	23.623	23.77	0.14	I_1:100	22.59	**22.84**	**0.50**
SwB_1:100	7.1069	7.05	0.06	1:100	22.859			SwB_1:100	20.801	**20.65**	**0.18**
1:100	8.2559			1:1000	26.383			1:100	20.374		
1:1000	11.203			1:10.000	29.629			1:1000	23.489		
1:10.000	15.596			1:100.000	32.01			1:10.000	26.631		
1:100.000	18.898			I_1:100	23.13			1:100.000	29.867		
1:1.000000	21.99			SwB_1:100	23.807			1:1.000000	32.746		
I_1:100	7.5724			1:100	23.025			I_1:100	23.415		
SwB_1:100	7.0519			1:1000	26.727			SwB_1:100	20.709		
1:100	8.1095			1:10.000	29.476			1:100	20.18		
1:1000	11.856			1:100.000	31.954			1:1000	22.889		
1:10.000	15.926			I_1:100	23.059			1:10.000	25.92		
1:100.000	18.851			SwB_1:100	23.894			1:100.000	29.152		
1:1.000000	22.431							1:1.000000	31.861		
I_1:100	7.601							I_1:100	22.506		
SwB_1:100	6.9897							SwB_1:100	20.445		
								-RT_1:100	33.228		
								-RT_1:100	33.271		
**Ref. gene stability (mean of NI, I, SwB)**		7.72	0.30			23.22	0.03				

I= induced with 1 mM IPTG, 107 nM aTc (= filamentous samples), SwB = switch back samples (previously treated with 1 mM IPTG, 107 nM aTc and 100 nM AHL)
