# Supplementary material for: Filamentation and restoration of normal growth in Escherichia coli using a combined CRISPRi sgRNA/antisense RNA approach
Source: PLoS One. 2018 Sep 11;13(9):e0198058. doi: 10.1371/journal.pone.0198058 (PMC6133276; doi:10.1371/journal.pone.0198058)
Supplement: S6 Table — From the obtained Cq values (see S4 Table), the amplification efficiencies for reference genes rrsB and cysG were extracted from the linear fit equations. (PDF) [file pone.0198058.s016.pdf]

	**equation**	**amplification efficiency**
rrsB	y = -3.6 + 0.7, R^2>0.99	90%
cysG	y = -3.0 + 17, R^2>0.99	115%
